# Macrophages-aPKC_ɩ_-CCL5 Feedback Loop Modulates the Progression and Chemoresistance in Cholangiocarcinoma

**DOI:** 10.1186/s13046-021-02235-8

**Published:** 2022-01-15

**Authors:** Tao Yang, Zhengdong Deng, Lei Xu, Xiangyu Li, Tan Yang, Yawei Qian, Yun Lu, Li Tian, Wei Yao, Jianming Wang

**Affiliations:** 1 Department of Biliary and Pancreatic Surgery/Cancer Research Center Affiliated Tongji Hospital, Tongji Medical College, Huazhong University of Science and Technology, Wuhan, 430030 Hubei China; 2 Department of Hepatobiliary Surgery, Renmin Hospital of Wuhan University, Wuhan, 430060 Hubei China; 3 School of Pharmacy, Tongji Medical College, Huazhong University of Science and Technology, Wuhan, 430022 Hubei China; 4 Department of General Surgery, Jiangsu Province Hospital and Nanjing Medical University First Affiliated Hospital, Nanjing, 210009 Jiangsu China; 5 Department of Oncology Affiliated Tongji Hospital, Tongji Medical College, Huazhong University of Science and Technology, Wuhan, 430030 Hubei China; 6 Affiliated Tianyou Hospital, Wuhan University of Science & Technology, Wuhan, 430064 China

**Keywords:** Cholangiocarcinoma, Positive Feedback Loop, Macrophages, aPKC_ɩ_, Chemoresistance

## Abstract

**Background:**

Recent data indicated that macrophages may mutually interact with cancer cells to promote tumor progression and chemoresistance, but the interaction in cholangiocarcinoma (CCA) is obscure.

**Methods:**

10x Genomics single-cell sequencing technology was used to identified the role of macrophages in CCA. Then, we measured the expression and prognostic role of macrophage markers and aPKC_ɩ_ in 70 human CCA tissues. Moreover, we constructed monocyte-derived macrophages (MDMs) generated from peripheral blood monocytes (PBMCs) and polarized them into M1/M2 macrophages. A co-culture assay of the human CCA cell lines (TFK-1, EGI-1) and differentiated PBMCs-macrophages was established, and functional studies in vitro and in vivo was performed to explore the interaction between cancer cells and M2 macrophages. Furthermore, we established the cationic liposome-mediated co-delivery of gemcitabine and aPKC_ɩ_-siRNA and detect the antitumor effects in CCA.

**Results:**

M2 macrophage showed tumor-promoting properties in CCA. High levels of aPKC_ɩ_ expression and M2 macrophage infiltration were associated with metastasis and poor prognosis in CCA patients. Moreover, CCA patients with low M2 macrophages infiltration or low aPKC_ɩ_ expression benefited from postoperative gemcitabine-based chemotherapy. Further studies showed that M2 macrophages-derived TGFβ1 induced epithelial-mesenchymal transition (EMT) and gemcitabine resistance in CCA cells through aPKC_ɩ_-mediated NF-κB signaling pathway. Reciprocally, CCL5 was secreted more by CCA cells undergoing aPKC_ɩ_-induced EMT and consequently modulated macrophage recruitment and polarization. Furthermore, the cationic liposome-mediated co-delivery of GEM and aPKC_ɩ_-siRNA significantly inhibited macrophages infiltration and CCA progression.

**Conclusion:**

our study demonstrates the role of Macrophages-aPKC_ɩ_-CCL5 Feedback Loop in CCA, and proposes a novel therapeutic strategy of aPKC_ɩ_-siRNA and GEM co-delivered by liposomes for CCA.

**Supplementary Information:**

The online version contains supplementary material available at 10.1186/s13046-021-02235-8.

## Background

Cholangiocarcinoma (CCA) is one of the most highly malignant and lethal cancers with limited overall survival and is notorious for its rapid progression, early metastasis and therapeutic resistance. Although radical resection is the most effective approach for prolonging long-term survival, more than two-thirds of patients lack operative opportunities for locally advanced or distant metastatic tumors. Moreover, even with aggressive surgical treatment, the 5-year survival rates of CCA patients remain unsatisfactory [1, 2]. Gemcitabine (GEM) based chemotherapy is the first-line of care treatment for CCA, but it does not significantly improve the overall survival of CCA patients [3, 4]. Therefore, there is a desperate need to identify the mechanism of chemoresistance and develop improved therapeutic strategies for CCA patients.

The tumor microenvironment (TME) plays a significant role in promoting cancer progression, invasion, metastasis and chemoresistance [5]. Macrophages in the TME, namely tumor-associated macrophages (TAMs), exhibit different phenotypes and functional features owing to pathogen or cytokine stimulation. TAMs often display an alternatively activated (M2) phenotype and enhance tumor malignancy in the majority of cases [6], and cancer cells can actively modulate the recruitment and activation of macrophages to enhance tumor growth and metastasis [7, 8]. CCA is characteristically marked by a highly desmoplastic and hypovascularized microenvironment [9]. The prognostic and clinical significance of TAMs has previously been reported in CCA, and TAMs are correlated with poor prognosis and dismal survival outcomes [10]. Furthermore, Kitano et al. [11] identified a risk signature, derived from the integration of intratumoral neutrophils, macrophages, CD8^+^T cells and Tregs, related to GEM chemoresistance in CCA patients. Noteworthy, accumulated evidence has shown that cancer cells and stromal cells interact in tumor microenvironment to regulate tumor growth and progression and offer a potential treatment strategy for cancer patients [12]. Ljichi et al. [13] reported that targeting tumor-stromal interactions could reduce chemoresistance and improve survival in a mouse model of pancreatic ductal adenocarcinoma. However, the role and mechanism of the crosstalk between TAMs and cancer cells in CCA are still unclear.

It has been demonstrated that epithelial-mesenchymal transition (EMT), a process whereby differentiated epithelial cells lose their polarity and acquire a mesenchymal phenotype, is predominantly associated with chemoresistance, cancer stem cells and the immune microenvironment [14]. We previously demonstrated that atypical protein kinase C iota (aPKCι) promotes EMT and induces immunosuppression in CCA, suggesting that aPKCι may play a pivotal role in the interaction between cancer cells and the TME [15]. Herein, we provide the first report of a positive feedback loop between macrophages and cancer cells that promotes CCA progression and chemoresistance. Our results indicate that M2 macrophages secrete TGFβ1 to induce cancer cell EMT and chemoresistance through the aPKC_ɩ_-NF-κB signaling pathway in CCA. Reciprocally, CCL5 levels are significantly increased in the supernatant of CCA cells that undergo aPKC_ɩ_-induced EMT and consequently modulate the recruitment and activation of macrophages.

Combination therapy with siRNAs and chemotherapeutic drugs has been considered as an alternative option to enhance the anticancer efficiency [16, 17]. Other groups, as well as us, have investigated whether the co-delivery of chemotherapeutic drugs and siRNAs by liposome- or nanoparticle-based systems could be a promising strategy for overcoming chemoresistance in breast cancer and pancreatic cancer [16, 18]. Given the crucial roles of aPKC_ɩ_, we prepared cationic liposomes to co-deliver GEM and aPKC_ɩ_-siRNA to improve the chemotherapeutic responses and treatment efficacy of CCA.

In the study, we describe the interaction between macrophages and CCA cells, and provide a novel combination therapy strategy with aPKC_ɩ_-siRNA and GEM using a liposome-based drug delivery system for CCA patients.

## Results

### The clinical significance of macrophages infiltration and aPKCι in human CCA

**Fig. 1:**
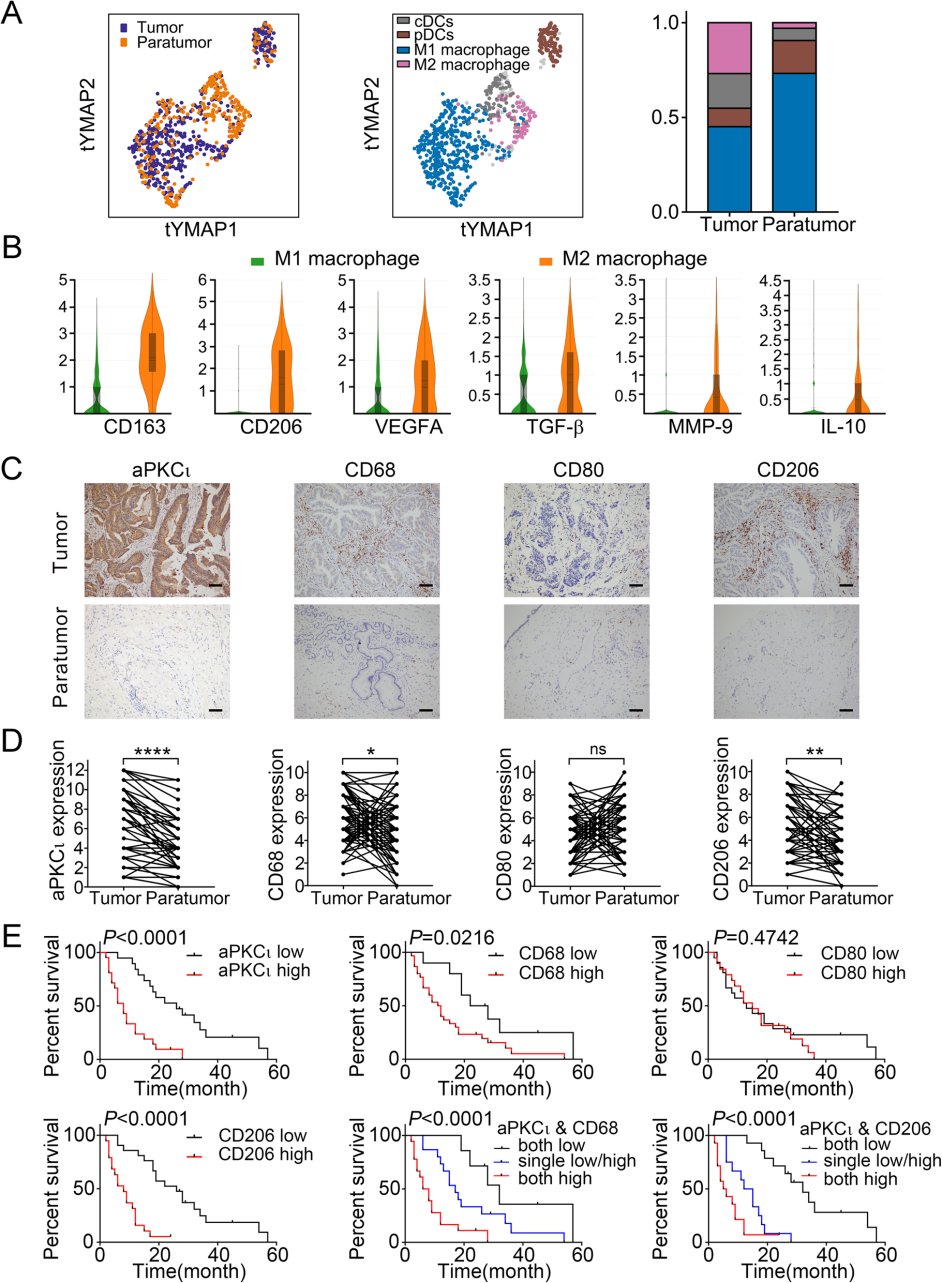
The clinical significance of macrophages infiltration and aPKCι in human CCA. **A**. Reclassification of Myeloid Immune Cell in Cholangiocarcinoma based on Single Cell RNA Sequencing. **B**. The expression of TAMs markers and some cancer-promoting genes in each Macrophages subtypes. **C**. IHC was used to examine the levels of aPKC_ɩ_ (1:200) and macrophage markers such as CD68 (1:150), CD80 (1:500), and CD206 (1:400) in 70 paired CCA and paratumor tissues. Representative images are shown. Scale bar, 20 μm. **D**. Quantification of aPKC_ɩ_ and macrophage markers expression level in paired CCA and paratumor tissues. **E**. Kaplan-Meier analysis indicating the correlation between the prognosis of CCA patients and the expression of aPKC_ɩ_, CD68, CD80 and CD206

To better understand the role of macrophages in the cholangiocarcinoma tumor environment (TME), we employed the 10x Genomics single-cell sequencing technology to reveal the phenotype and function of myeloid immune cells in human cholangiocarcinoma. As shown in Fig. 1A, the myeloid immune cells in CCA tumor and paratumor tissues are mainly divided into 4 cell subtypes after dimensionality reduction cluster analysis: M1 macrophage subtype, M2 macrophage subtype, a classical dendritic cell subtype and a plasmacytoid dendritic cell subtype. We found that the proportion of M1 macrophages in tumor tissue was lower than that in paratumor tissue (42.18% vs 70.09%), whereas the proportion of M2 macrophages was higher in tumor tissue (25.09% vs 2.78%). Despite a greater proportion of M1 macrophages in the TME, M2 macrophages expressed higher gene level of CD163, MRC1 (CD206), TGF-β, IL10, VEGFA, and MMP9, which exhibited stronger tumor-promoting ability (Fig. 1B).

To investigate the clinical significance of macrophage infiltration and aPKC_ɩ_ expression in human CCA, we examined aPKCι and TAMs markers (macrophage: CD68; M1 macrophage: CD80; M2 macrophage: CD206) expressed in human CCA by immunohistochemistry (IHC), Western blotting (WB), and quantitative real-time polymerase chain reaction (qRT-PCR). The IHC analysis showed that staining of aPKCι, CD68, and CD206 was enriched in CCA tissues than pair-matched paratumor tissues (Fig. 1C-D). Consistent results were verified by WB and qRT-PCR (Supplementary Fig. 1A-B). Then, we analyzed the association between aPKC_ɩ_ and TAMs markers in CCA specimens. Pearson correlation analyses of the above IHC results confirmed that expression of aPKC_ɩ_ was positively correlated with CD68 (r = 0.4128, *P* = 0.0004) and CD206 (r = 0.5489, *P* < 0.0001). However, there was no significant correlation between aPKC_ɩ_ and CD80 (r = 0.0540, *P* = 0.6569) (Supplementary Fig. 1C).

We further explored the correlation of aPKCι expression and macrophage infiltration with the clinicopathological characteristics and prognosis in patients with CCA. Notably, overexpression of aPKC_ɩ_ and CD206 was remarkably associated with lymph node metastasis (χ^2^ = 6.005, 4.086; *P* = 0.014, *P* = 0.043, respectively), tumor-node-metastasis (TNM) stage III-IV (χ^2^ = 6.740, 12.899; *P* = 0.009, *P* < 0.001), and moderate/poor differentiation (χ^2^ = 3.994, 4.073; *P* = 0.046, *P* = 0.044). High level of CD68 was just related to lymph node metastasis (χ^2^ = 4.076; *P* = 0.044) and tumor-node-metastasis (TNM) stage III-IV (χ^2^ = 5.871; *P* = 0.015), while CD80 was not associated with clinicopathological characteristics (Table 1). Moreover, a Kaplan-Meier analysis revealed that patients with high level of aPKCι, CD68 or CD206 rather than CD80 displayed worse overall survival (OS). Prognosis was also statistically associated with the co-expression of aPKCι and CD68/CD206 (Fig. 1E). Multivariate Cox regression analyses indicated that aPKCι and CD206 were independent prognostic factors for OS in CCA patients (Table 2). These data imply that aPKCι and CD206^+^ macrophage (M2 macrophage), but not CD80^+^ macrophage (M1 macrophage), may contribute to the progression and dismal prognosis of CCA.

**Table 1:** Correlation Between aPKC_ɩ_, CD68, CD80, CD206, NF-κB, and Clinicopathologic Characteristics in 70 CCA Patients

Group	n	aPKC_ɩ_		*P*	CD68		*P*	CD80		*P*	CD206		*P*	NF-κB		*P*
		Low	High		Low	High		Low	High		Low	High		Low	High	
Age (years)																
≤ 60	37	19	18	0.208	12	25	0.449	18	19	0.602	19	18	0.622	19	18	0.316
> 60	33	12	21		8	25		14	19		15	18		13	20	
Gender																
Male	36	17	19	0.611	10	26	0.880	12	24	0.089	16	20	0.477	17	19	0.794
Female	34	14	20		10	24		20	14		18	16		15	19	
Lymphoid nodal status																
No	45	24	21	0.041	16	29	0.044	20	25	0.866	25	18	0.043	28	15	<0.001
Yes	25	7	18		4	21		12	13		9	18		4	23	
TNM staging																
I-II	33	20	13	0.009	14	19	0.015	16	17	0.819	24	9	<0.001	25	8	<0.001
III-IV	37	11	26		6	31		16	19		11	26		7	30	
Differentiation																
Well	27	16	11	0.046	9	18	0.485	11	16	0.508	17	10	0.044	16	11	0.071
Moderate/Poorly	43	15	28		11	32		21	22		17	26		16	27

**Table 2:** Univariate and multivariate analyses for predictors of overall survival

Vriables	Overall survival					
	Univariate analysis			Multivariate analysis		
	HR	95%CI	***P*** value	HR	95%CI	***P*** value
Age (<60 vs ≥60)	1.574	0.779-3.178	0.206			
Gender (male vs female)	0.731	0.371-1.440	0.365			
Lymph node metastasis (no vs yes)	5.004	2.258-11.087	<0.001	10.933	4.133-14.481	0.027
TNM stage (I/II vs III/IV)	3.812	1.783-8.147	0.001			
Differentiation (well vs moderate/poor)	7.726	2.644-22.582	<0.001	8.368	2.403-29.145	0.001
aPKC_ɩ_ expression (low vs high)	4.329	2.000-9.368	<0.001	4.766	1.549-14.661	0.006
CD68 expression (low vs high)	2.529	1.094-5.844	0.030			
CD80 expression (low vs high)	1.272	0.646-2.503	0.486			
CD206 expression (low vs high)	5.981	2.567-13.936	<0.001	3.526	1.268-9.808	0.016

*HR* hazard ratio, *CI* confidence interval

### M2 macrophages induce aPKCι-mediated CCA cell chemoresistance to GEM

**Fig. 2:**
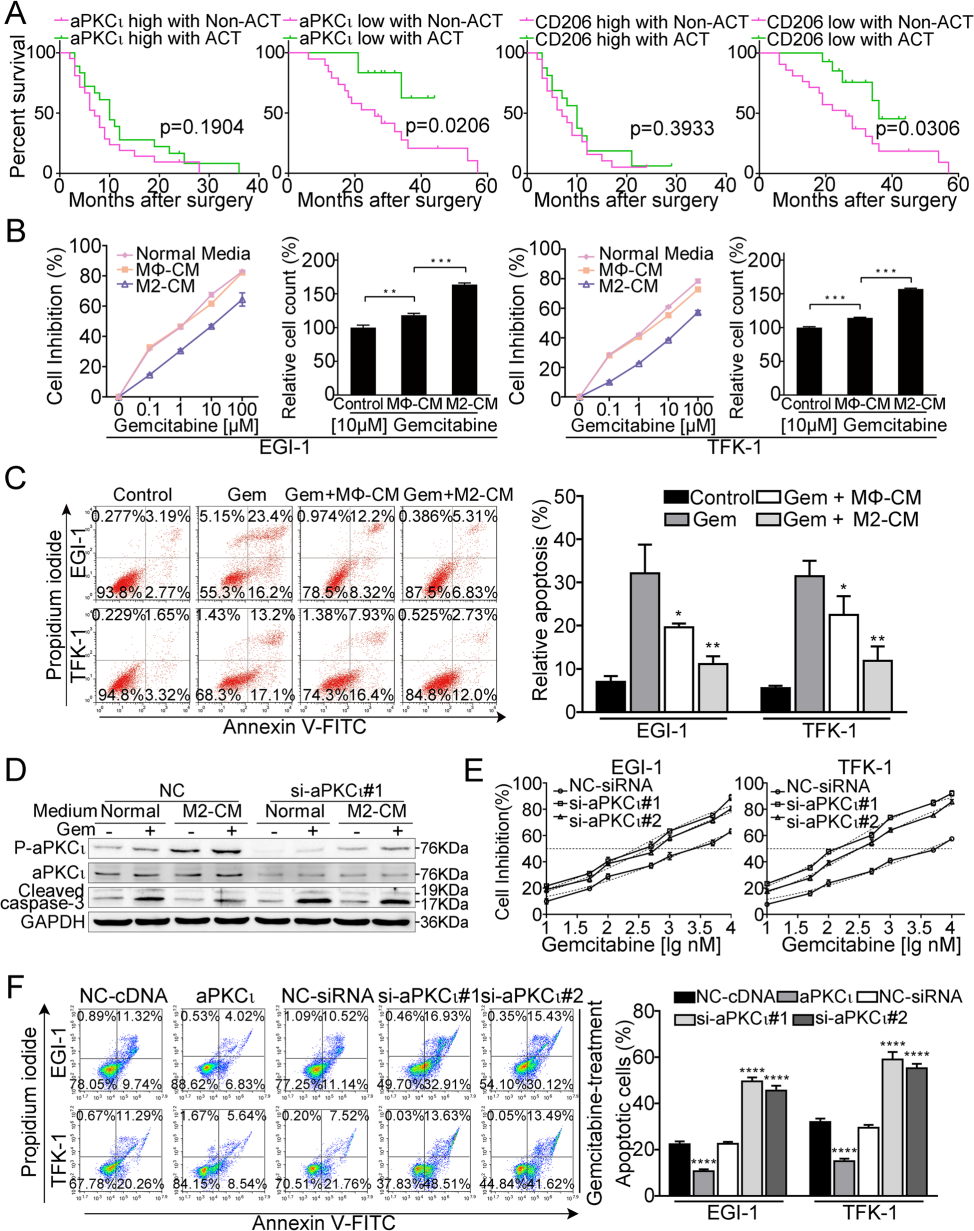
M2 macrophages induce aPKCι-mediated CCA cell chemoresistance to GEM. **A**. Overall survival rates of CCA patients with the different expression levels of aPKC_ɩ_ and CD206 treated with GEM-based chemotherapy or not after surgery were compared using Kaplan-Meier analysis. **B**. The CCK-8 assay was used to detect the sensitivity of CCA cells to GEM under different conditions: normal media, MΦ-CM and M2-CM. Relative number of CCΑ cells treated with 10 μΜ GEM in different conditioned media as indicated for 48 h. **C**. CCA cells were incubated in different conditions as indicated in the presence or absence of 10 μM GEM for 48 h, and the percentage of apoptotic cells was analyzed by FACS. **D**. WB of p-aPKC_ɩ_ and cleaved caspase-3 (WB of CCA cells transfected with scrambled control or si-aPKC_ɩ_#1 and treated with or without GEM in normal media or M2-CM for 48 h). **E**. IC50 of gemcitabine in the indicated cells. Each bar represents the mean ± SD of three independent experiments. **F**. Annexin V-FITC and PI staining of the indicated cells treated with gemcitabine (10 μM) for 48 h. Each bar represents the mean ± SD of three independent experiments

To study the contribution of M2 macrophages and aPKCι to chemoresistance, we investigated the efficacy of postoperative GEM-based chemotherapy in CCA patients. As shown in Supplementary Fig. 2A, chemotherapy did not provide apparent survival benefit in CCA patients. However, we found patients with high aPKCι expression exhibited no response to postoperative chemotherapy, while patients with low aPKCι expression responded well (Fig. 2A). Consistently, patients with low CD206^+^ macrophage infiltration displayed a favorable outcome after postoperative chemotherapy, whereas no apparent benefit was found in patients with high CD206^+^ macrophage infiltration (Fig. 2A). These findings indicate that aPKCι and M2 macrophage infiltration are associated with chemoresistance in CCA.

To identify the effects of M2 macrophages on CCA cells, we applied a model of macrophage polarization involving the differentiation of peripheral blood mononuclear cells (PBMCs) for further analysis (Supplementary Fig. 2B). Flow cytometry and RT-PCRs were employed to analyze the phenotype of macrophages. The M2 macrophage characteristics were confirmed by the reduced expression of M1 markers (CD80 and IL-12) and the elevated expression of M2 markers (CD206 and IL-10) (Supplementary Fig. 2C-D). Consequently, we examined whether M2 macrophages could protect CCA cells from GEM chemotherapy. As shown in Fig. 2B, compared with control medium and MΦ-CM treatments, M2-CM treatment significantly reduced the sensitivity of CCA cells to GEM. Next, FACS, which was employed to detect apoptosis indicated that M2-CM treatment notably reduced the apoptosis of CCA cells induced by 10 μM GEM relative to control medium and MΦ-CM treatments (Fig. 2C). Previous studies have shown that elevated aPKCι expression provides resistance to drug-induced apoptosis [19]. To verify whether aPKCι is involved in the M2-CM-mediated protection of CCA cells against GEM-induced apoptosis, we established aPKCι-deficient CCA cell lines by transfection with aPKCι-siRNA (Supplementary Fig. 2E). WB analysis showed that the protein level of cleaved caspase-3 in cells treated with GEM plus M2-CM was markedly decreased compared to that in cells treated with GEM plus control medium. aPKCι depletion resulted in loss of the M2-CM-mediated protective effect (Fig. 2D). Interestingly, the level of phosphorylated-aPKC_ɩ_ was significantly increased in CCA cells after M2-CM treatment, while the level of aPKC_ɩ_ was not affected (Fig. 2D). We further found that the IC50 value for GEM was drastically increased in aPKCι-overexpression CCA cells, but decreased in aPKCι-silenced cells (Fig. 2E and Supplementary Fig. 2F). Moreover, FACS was employed to validate the anti-apoptosis role of aPKC_ɩ_ in CCA cells and found that upregulated aPKCι significantly reduced the GEM-induced apoptosis rates of CCA cells (Fig. 2F). However, overexpressing or silencing aPKCι only resulted in a slight change in the apoptosis rate of CCA cells without GEM treatment (Supplementary Fig. 2G). These results indicate that aPKCι may play a key role in M2 macrophage-induced CCA cell GEM resistance.

### aPKCι mediates NF-κB activation contributing to M2 macrophages-induced chemoresistance

**Fig. 3:**
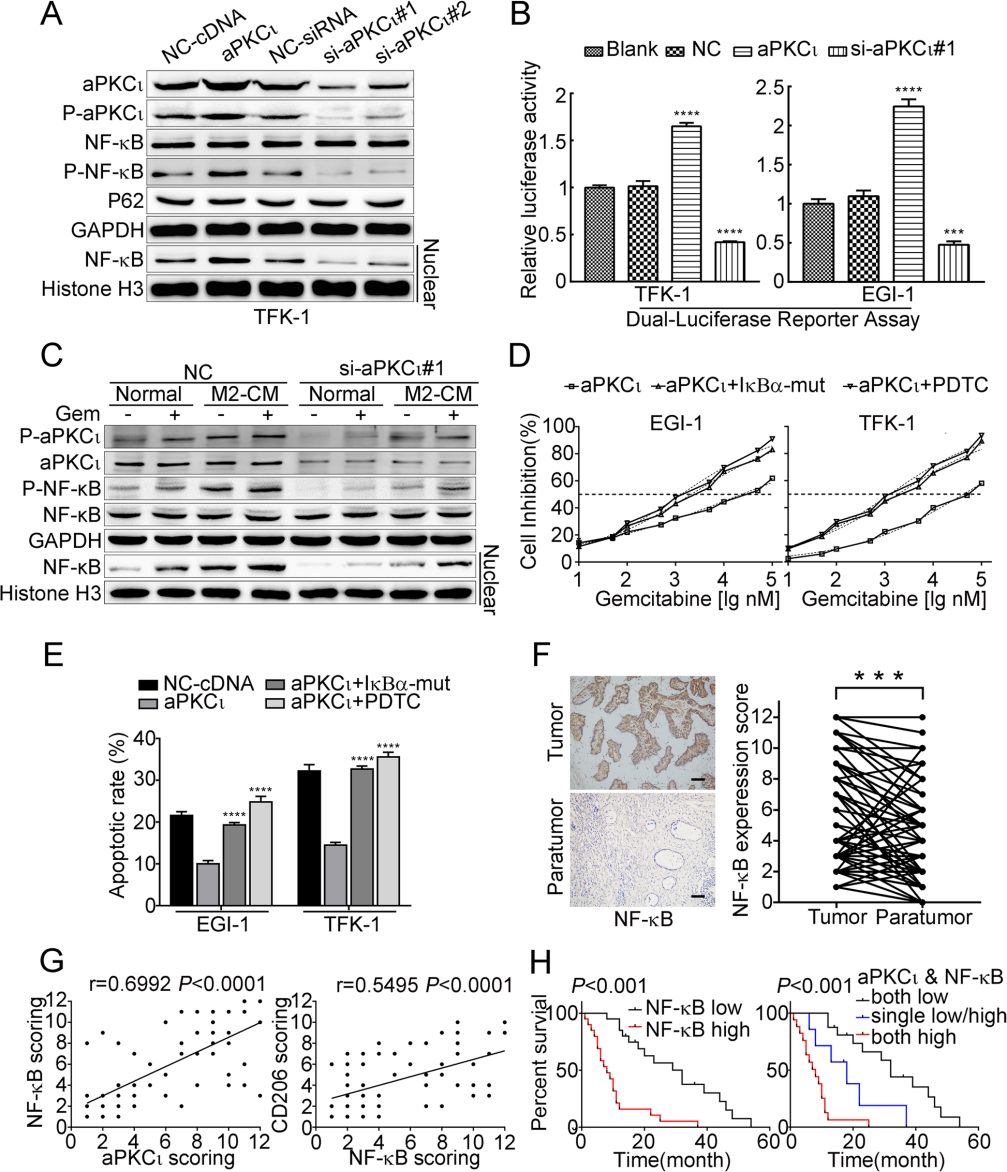
aPKCι mediates NF-κB activation to contribute to M2 macrophages-induced chemoresistance. **A**. WBs were used to detect the expression of p-aPKC_ɩ_, P62, p-NF-κB and test the NF-κB (p65) nuclear translocation in the indicated cells. CCA cells were transfected with the empty vector as a negative control (NC) and cells without any treatment were used as blank control (aPKC_ɩ_). **B**. NF-κB luciferase reporter activity was detected by Dual-luciferase reporter assay. **C**. WB for the indicated proteins of TFK-1 cells transfected with scrambled control or si-aPKC_ɩ_#1 and treated with normal media or M2-CM for 6 h in the absence or presence of 10 μM GEM. **D**. IC50 of gemcitabine in the aPKC_ɩ_^+^ cells transfected with vector, IκBα-mut, or treated with an NF-κB inhibitor (PDTC). Each bar represents the mean ± SD of three independent experiments. **E**. Annexin V-FITC and PI staining of the indicated cells treated with gemcitabine (10 μM) for 48 h. **F**. IHC was used to examine the levels of nuclear NF-κB (1:200) in 40 paired CCA and paratumor tissues. Representative images are shown (left). Scale bar, 20 μm. Quantification of the nuclear NF-κB expression level in paired CCA and paratumor tissues (right). **G**. Linear regression was used to analyze the correlations between nuclear NF-κB with aPKC_ɩ_ and CD206. **H**. Kaplan−Meier analysis indicating the correlation between nuclear NF-κB expression and overall survival in patients with CCA (left). The overall survival curves from CCA patients with co-expression of aPKC_ɩ_ and nuclear NF-κB was analyzed by Kaplan–Meier (right)

Growing evidences have demonstrated that aPKCι activates NF-κB signaling in multiple tumor types [20–22] and that NF-κB is a major transcription factor associated with the immune microenvironment [23, 24], chemoresistance [25] and EMT [26, 27]. To further explore the mechanism by which aPKC_ɩ_ promotes CCA cells survival, we investigated the role of the NF-κB signaling pathway. We found that overexpressing aPKCι induced p65 phosphorylation and nuclear translocation (Fig. 3A and Supplementary Fig. 3A) and enhanced NF-κB transcriptional activity (Fig. 3B). Subsequently, WB was employed to validate that aPKCι mediates NF-κB activation in M2 macrophage-induced GEM resistance. We found that M2-CM induced aPKCι phosphorylation and NF-κB activation in the presence of GEM, while aPKC_ɩ_-siRNA treatment attenuated these effects (Fig. 3C). Furthermore, we assessed whether the NF-κB signaling was required for aPKC_ɩ_-induced chemoresistance. We blocked the NF-κB signaling pathway by pharmacologically employing pyrrolidinedithiocarbamate ammonium (PDTC 50umol/L) or genetically exerting a dominant negative mutant of IκBα. Inhibition of NF-κB signaling enhanced the GEM-induced apoptosis rates and reduced the IC50 value of GEM in aPKC_ɩ_-overexpressing CCA cells (Fig. 3D-E). Anchorage-independent growth of aPKC_ɩ_-overexpressing CCA cells, treated with GEM (10μM), was suppressed in the presence of PDTC (Supplementary Fig. 3B).

Based on these findings, the clinical significance of nuclear NF-κB (P65) expression was characterized in human CCA. The IHC analysis showed that staining of nuclear NF-κB was enriched in CCA tissues than pair-matched paratumor tissues (Fig. 3F). Then, we analyzed the association between NF-κB and aPKC_ɩ_/CD206 in CCA specimens. Pearson correlation analyses of the IHC results confirmed that expression of nuclear NF-κB (P65) was significantly correlated with aPKC_ɩ_ (r = 0.6992, *P* < 0.0001) and CD206 (r = 0.5495, *P* < 0.0001) (Fig. 3G). Moreover, the positive immunoreactivity of nuclear NF-κB was significantly associated with lymph node metastasis (χ^2^ = 16.911, *P* < 0.001) and tumor-node-metastasis (TNM) stage III-IV (χ^2^ = 22.707, *P* < 0.001) as shown in Table 1. The group with low nuclear NF-κB expression had better prognosis (Fig. 3H). Importantly, the prognosis was also statistically associated with the co-expression of aPKCι and CD206 (Fig. 3H and Supplementary Fig. 3C). These results indicated that aPKCι mediates NF-κB activation to contribute to M2 macrophages-induced GEM resistance.

In terms of a mechanism, previous research has confirmed that the aPKC_ɩ_ could bind to P62 through a PB1-PB1 domain interaction that regulates NF-κB activation [22]. Therefore, we assessed whether this mechanism exists in CCA. We performed Co-IP experiments and found that aPKC_ɩ_ immunoprecipitated with P62, and in turn, P62 was detected in the aPKCι-immunoprecipitates (Supplementary Fig. 3D upper). To determine the molecular surfaces through which the aPKCι and P62 interaction occur, we constructed Flag-tagged wild-type and site-specific mutants (aPKC_ɩ_-D72A, P62-K7A) of these proteins based on previously reported research findings [28–30]. IP experiments showed that the interaction of aPKC_ɩ_ with P62 requires the wild-type PB1 domain, and the D72A mutation in the PB1 domain of aPKC_ɩ_ abolished the interaction with P62 (Supplementary Fig. 3D bottom). In addition, WBs and luciferase reporter gene assays showed that NF-κB phosphorylation and transcriptional activity are regulated by P62. The K7A mutation in the PB1 domain of P62 abolished the WT effects (Supplementary Fig. 3E-F). This observation is similar to that of Wooten et al. [31] These results provide evidence that aPKCι may regulate NF-κB activation by interacting with P62.

### TAMs-derived TGFβ1 induce CCA cell EMT via the aPKC_ɩ_ and NF-κB activation

**Fig. 4:**
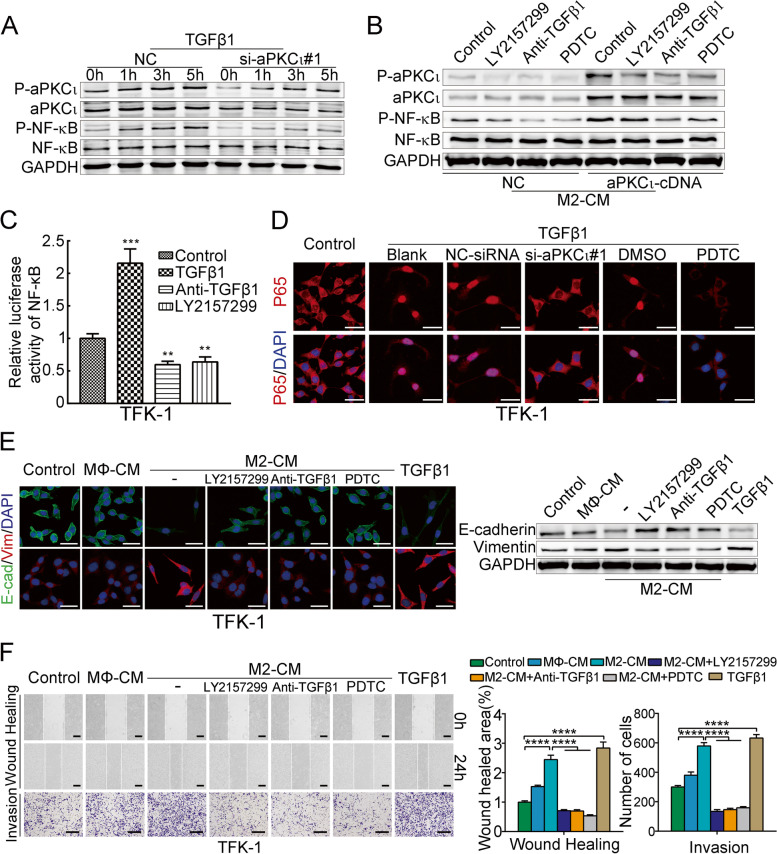
TAMs-derived TGFβ1 induce CCA cell EMT via the aPKC_ɩ_ and NF-κB activation. **A**. WBs was used to detect the activation of aPKC_ɩ_ and NF-κB in CCA cells transfected with control-siRNA or aPKC_ɩ_-siRNA after TGFβ1 treatment for the indicated times. **B**. WBs were employed to detect the activation of aPKC_ɩ_ and NF-κB in CCA cells transfected with aPKC_ɩ_ cDNA or control-cDNA after treatment with CM from M2 macrophages alone or those treated with a TGFβR1 inhibitor (LY2157299), an anti-TGFβ1 neutralizing antibody, or pyrrolidinedithiocarbamate ammonium (PDTC). **C**. NF-κB luciferase reporter NF-κB activity in the indicated cells, either treated with TGFβ1 for 5 h or treated with or without LY2157299 (2 μM) or a neutralizing anti-TGFβ1 antibody (2 μg/mL) for 5 h. **D**. Confocal fluorescence microscopy of p65/DAPI staining in TFK-1 cells treated with or without TGFβ1 together with PDTC or aPKC_ɩ_-siRNA treatment. Scale bar, 20 μm. **E**. Immunofluorescence and WB were used to detect E-cadherin and vimentin expression in CCA cells treated with or without CM from MΦ macrophages (MΦ-CM), M2-CM alone or with LY2157299, an anti-TGFβ1 neutralizing antibody, PDTC, or recombinant human TGFβ1. Scale bar, 20 μm. **F**. Wound healing and invasion assays of CCA cells treated as described in (E). Scale bar, 200 μm (mean ± SD, *n* = 3; *P < 0.05, **P < 0.01, ***P < 0.001 and ****P < 0.0001; *P* values were obtained using two-tailed Student’s t tests)

TGFβ1 is well known to facilitate tumor formation and development [32]. Coincidentally, it is also one of the major cytokines secreted by TAMs^5^. Consistent with the results of the 10x Genomics single-cell sequencing technology in human cholangiocarcinoma, ELISA and qPCR showed that TGFβ1 was dramatically upregulated in intracellular and supernatant of M2 macrophages compared with MΦ/M1 macrophages (Supplementary Fig. 4A). Therefore, we hypothesized that M2 macrophages-derived TGFβ1 might contribute to the promotion of CCA cell EMT. We have previously demonstrated the critical role of aPKC_ɩ_ in TGFβ1-induced EMT in CCA cells [33]. Here, we found that TGFβ1 induced aPKC_ɩ_ and NF-κB phospho-activation in a time-dependent manner, whereas aPKC_ɩ_-siRNA treatment attenuated the effects (Fig. 4A). Moreover, the levels of p-aPKC_ɩ_ and p-NF-κB were increased in aPKC_ɩ_-transduced CCA cells treated with M2-CM, while anti-TGFβ1 neutralizing antibody or LY2157299 (a selective TGFβ receptor inhibitor) treatment reversed the above effects (Fig. 4B). Similarly, we found that TGFβ1 induced NF-κB transcriptional activity and nuclear translocation, and this response was dependent on aPKC_ɩ_, because aPKC_ɩ_ depletion through siRNA resulted in loss of the effect (Fig. 4C-D and Supplementary Fig. 4B).

We have found that aPKC_ɩ_ induces EMT-like protein expression [15] (Supplementary Fig. 4C), it was not surprising to see that the morphology of CCA cells treated with M2-CM changed from a broad elliptical shape to a long fusiform shape. The M2-CM-treated CCA cells had reduced expression of E-cadherin and increased expression of vimentin, which facilitates migration and invasiveness. However, anti-TGFβ1 neutralizing antibody or LY2157299 treatment suppressed the above EMT changes, TGFβ1 treatment alone acted as a positive control, suggesting that M2 macrophages-derived TGFβ1 induced CCA cell EMT (Fig. 4E-F and Supplementary Fig. 4D-E). Meanwhile, to further investigate whether NF-κB activation was essential for M2 macrophages-derived TGFβ1-induced CCA cells EMT, we employed PDTC to block the NF-κB signaling pathway. Strikingly, the inhibition caused reversal of TGFβ1-induced EMT, including up-regulation of E-cadherin, down-regulation of vimentin, as well as decreased capacity of cell invasion and migration (Fig. 4E-F and Supplementary Fig. 4D-E). Collectively, these data suggested that TAMs-derived TGFβ1 induces CCA cell EMT via the aPKC_ɩ_/NF-κB pathway.

### CCL5 secreted by aPKCι-induced mesenchymal-like CCA cells mediates the chemotactic migration and activation of macrophages

**Fig. 5:**
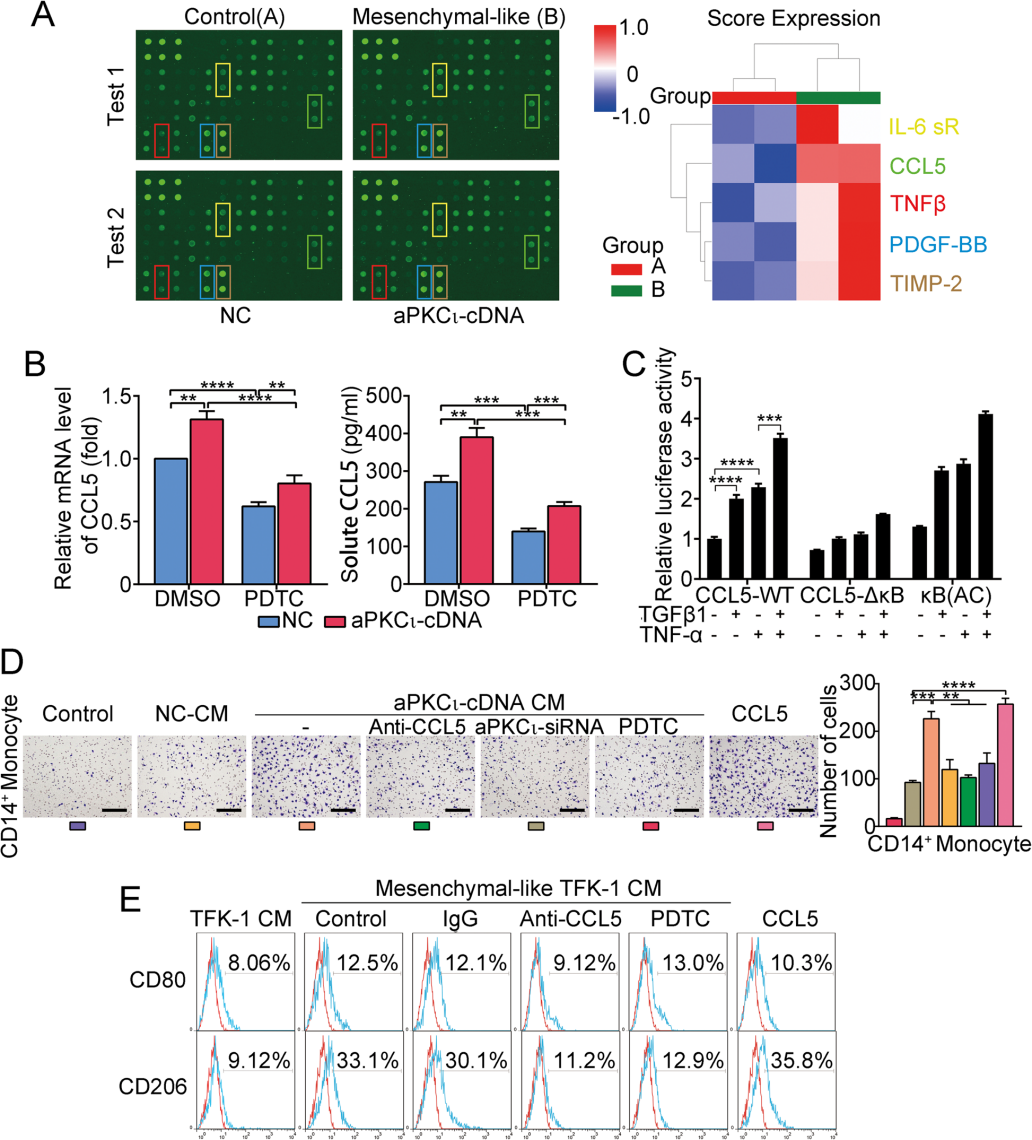
CCL5 secreted by aPKCι-induced mesenchymal-like CCA cells mediates the chemotactic migration and activation of macrophages. **A**. Cytokine array of the CM of mesenchymal-like TFK-1. The Euclidean average summarizing the relative signal intensity of the indicated cytokines is presented. **B**. qRT-PCR and ELISA showing the relative levels of CCL5 in mesenchymal-like or epithelial-like CCA cells treated without or with the NF-κB inhibitor PDTC. **C**. Luciferase reporter analysis of the NF-κB-mediated activation of CCL5 transcription. TFK-1 cells were transfected with luciferase reporter plasmids containing wild-type (CCL5-WT-Luc), NF-κB mutant (CCL5-ΔkB-Luc), or NF-κB response elements (κB-Luc, active control) in the absence or presence of TGF-β or TNF-α. **D**. Migration assay in CD14^+^ monocytes stimulated with or without CM from TFK-1 cells transfected with an empty vector (negative control, NC-CM), CM from mesenchymal-like TFK-1 cells (transfected with aPKCι-cDNA) alone or with anti-CCL5 neutralizing antibody, aPKCι-siRNA, PDTC, or recombinant human CCL5 treatment. Scale bar, 200 μm. **E**. Flow cytometry for expression of CD80/CD206 in macrophages treated with CM from mesenchymal-like TFK-1 cells in the presence or absence of control IgG, a CCL5 neutralizing antibody, PDTC, or recombinant human CCL5. The CM of TFK-1 cells transfected with empty vector was used as a negative control

To investigate whether mesenchymal-like CCA cells activate macrophages, we cocultured mesenchymal-like CCA cell lines by aPKCι transfection with PBMCs-induced macrophages. Flow cytometry showed that the proportions of M2 macrophages were significantly increased compared to macrophages cultured with the control group (Supplementary Fig. 5A). Moreover, cell migration assay revealed that CCA cells with aPKC_ɩ_ overexpression promote the CD14+ monocytes recruitment (Supplementary Fig. 5B). To understand how aPKCι-induced mesenchymal-like CCA cells exert their functions, we employed a human inflammation antibody array to identify the profile of cytokines secreted by mesenchymal-like TFK-1 cells. Interestingly, CCL5, which is a target gene of NF-κB [34] and an established chemoattractant for macrophages [35], was found among these cytokines (Fig. 5A). In agreement, ELISA, qRT-PCR and WB assays confirmed the increase of CCL5 production in mesenchymal-like CCA cells mediated by aPKCι (Fig. 5B and Supplementary Fig. 5C). Meanwhile, we found that NF-kB inhibition through PDTC resulted in loss of the aPKCι-mediated effect (Fig. 5B). To assess whether TGFβ1 and NF-κB transcription factors activate CCL5 promoter activity, luciferase reporter gene assays were performed using a CCL5 promoter with a mutated NF-κB binding site (Supplementary Fig. 5D). As expected, TGFβ1 treatment increased the transcriptional activity of CCL5, and the NF-κB binding site mutation in the CCL5 promoter attenuated this effect (Fig. 5C). On the basis of our data, we speculate that aPKCι/NF-κB/CCL5 signaling is involved in the macrophages recruitment and activation. To further verify our hypothesis we could show that CCL5 treatment enhanced M2 macrophage polarization and CD14^+^ monocytes recruitment, whereas targeting CCL5 with neutralizing antibodies potently abrogated the effects. Moreover, we also found that PDTC treatment attenuate these effects (Fig. 5D-E and Supplementary Fig. 5E). These findings indicated that TGFβ1 could induce the expression and secretion of CCL5 in CCA to mediate the chemotactic migration and activation of macrophages by aPKC_ɩ_/NF-κB signaling pathway.

Finally, we performed WB to analyze the mechanism of macrophages activation, and several classical signaling pathways involved in macrophage functions were evaluated. The results suggest that the activation of STAT3 signaling may be involved in CCL5-mediated macrophage recruitment and M2 polarization (Supplementary Fig. 5F).

### The macrophage-aPKC_ɩ_-CCL5 feedback loop promotes CCA growth and metastasis in vivo

**Fig. 6:**
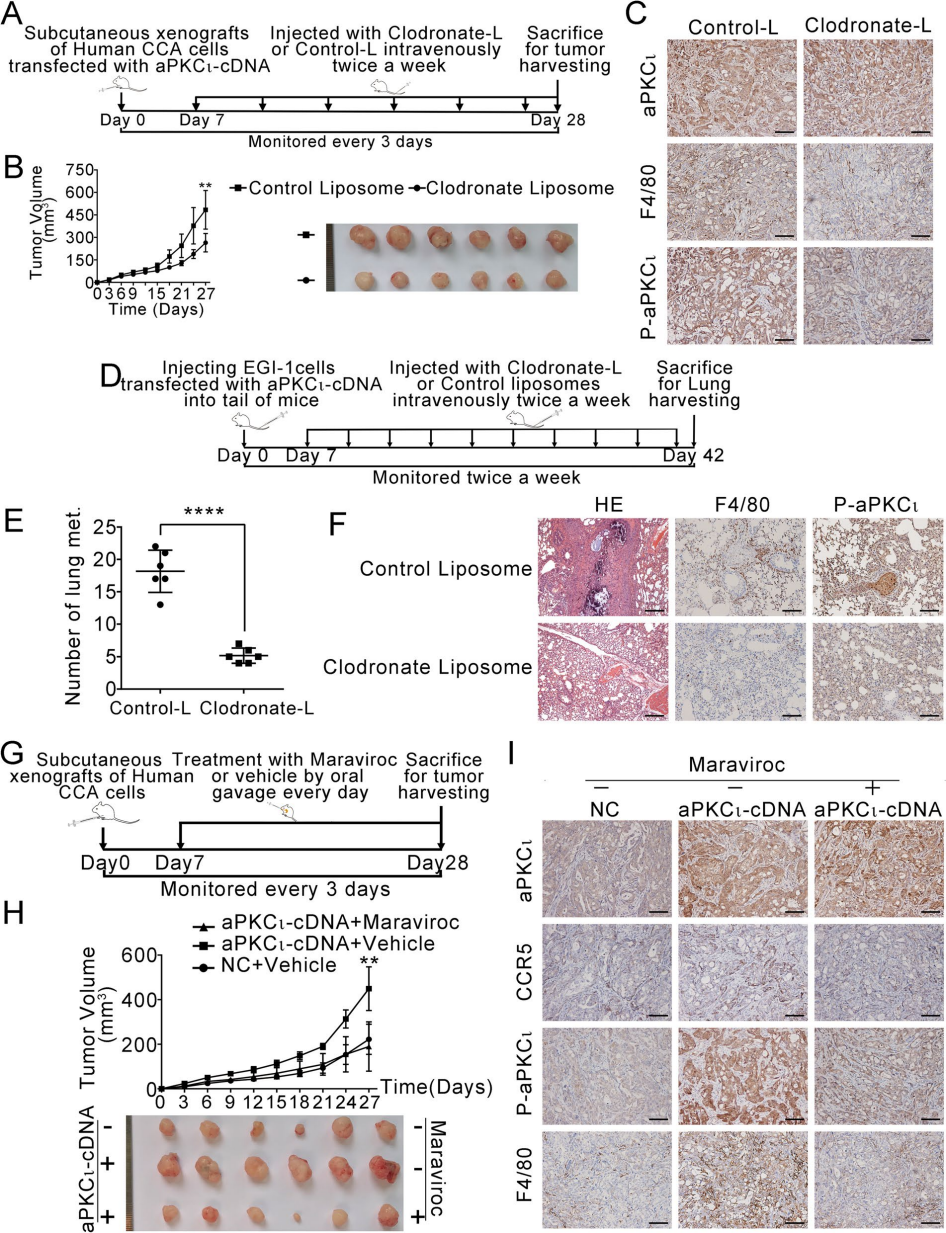
The macrophage-aPKC_ɩ_-CCL5 feedback loop promotes CCA growth and metastasis in vivo. **A**. Schematic for macrophage depletion in human CCA cell xenografts. **B**. The volume (left) and representative images (right) of xenograft tumors are shown from two different groups treated with clodronate liposomes or control liposomes. **C**. Representative images from tumor sample serial sections stained for aPKC_ɩ_, p-aPKC_ɩ_ and F4/80 are shown. Scale bar, 200 μm. **D**. Schematic for macrophage depletion in the lung metastasis models. **E**. The number of lung metastatic nodules was evaluated from different groups treated with or without clodronate liposomes. **F**. Haematoxylin-eosin (HE; left) and IHC (middle and right) were used to illustrate the expression of F4/80 and p-aPKC_ɩ_ in lung metastatic nodules. Scale bar, 200 μm. **G**. Schematic for macrophage recruitment in human CCA cell xenografts. **H**. The volume (upper) and representative images (bottom) of xenograft tumors are shown from three different groups as indicated. **I**. Representative images from xenograft tumor serial sections stained for aPKC_ɩ_, CCR5, p-aPKC_ɩ_ and F4/80 are shown. Scale bar, 200 μm

To investigate the crucial role of TAMs in the aPKCι-mediated progression of CCA in vivo, we established a xenograft model and lung metastasis model of human CCA according to the schematic shown in Fig. 6A and D. We found that the growth of xenografts was inhibited (Fig. 6B and Supplementary Fig. 6A) and the number of metastatic nodules was reduced after macrophages were selectively depleted (Fig. 6E). Notably, the depletion of macrophages resulted in a reduction in p-aPKCι expression (Fig. 6C, F and Supplementary Fig. 6B). These results suggest that the effect of TAMs on CCA progression in vivo may be mediated by the aPKCι signaling pathway. To further investigate the mechanism by which aPKCι regulates CCA development and the interaction between mesenchymal-like CCA cells and macrophages, we stably upregulated aPKCι expression in CCA cell lines. The macrophages recruitment experiment (Fig. 6G) demonstrated that aPKCι overexpression promoted tumor progression (Fig. 6H and Supplementary Fig. 6C). Furthermore, the number of F4/80^+^ macrophages in aPKCι-derived xenografts was higher than that in the negative control xenografts (Fig. 6I and Supplementary Fig. 6D). Because the CCL5-CCR5 axis is a major regulator of macrophage recruitment [36, 37], we investigated the role of aPKCι/CCL5 pathway in regulating macrophage recruitment in vivo. There was a greater infiltration of CCR5^+^ macrophages in aPKCι-derived tumors than control tumors, and antagonizing the CCL5-CCR5 axis significantly reversed this effect and decreased tumor development. These findings suggest that the effects of aPKCι overexpression on CCA progression are dependent on CCL5-mediated macrophage infiltration. Importantly, we observed that the reduction in macrophage recruitment potently inhibits the expression of p-aPKCι in CCA xenografts (Fig. 6I and Supplementary Fig. 6D). Combined with all the in vitro results, these findings suggest that aPKCι-induced CCL5 from mesenchymal-like CCA cells and TGFβ1 from TAMs form a positive feedback loop and promote CCA progression, and that aPKCι plays a key role in this process.

### Co-delivery of aPKCι-siRNA and GEM via liposomes for the effective treatment of CCA

**Fig. 7:**
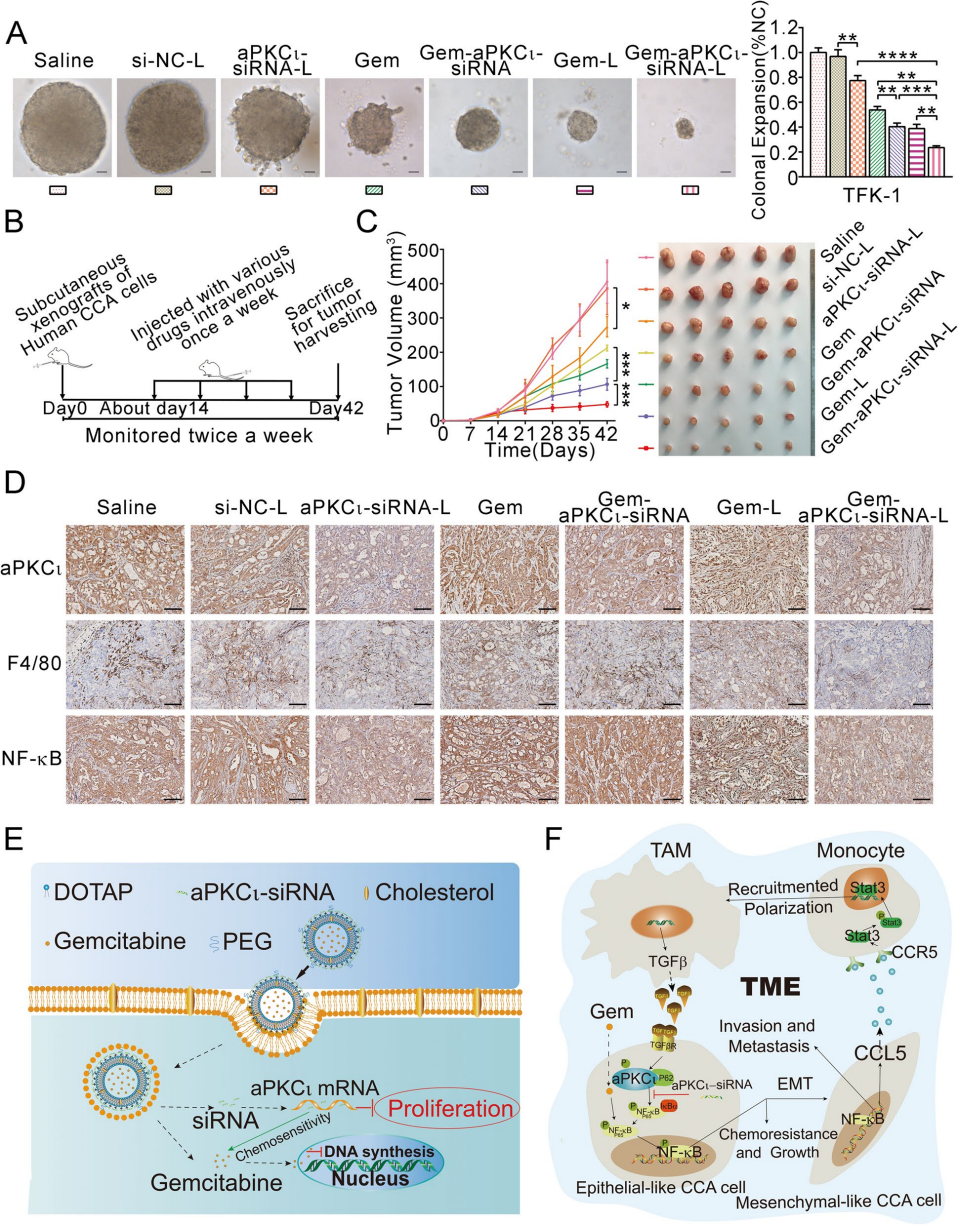
Co-delivery of aPKCι-siRNA and GEM via liposomes for the effective treatment of CCA. **A**. Anchorage-independent growth of CCA cells in soft agar in the presence of si-NC-L, aPKCι-siRNA-L, GEM, GEM-aPKCι-siRNA, GEM-L, GEM-aPKCι-siRNA-L compared with that of cells in the presence of PBS alone. **B**. Schematic demonstrating the method for the analysis of the antitumor effect in human CCA cell xenografts. **C**. The tumor volume (left) and representative images (right) of xenograft tumors are shown from different groups. Data represent the mean ± SEM. *P < 0.05, ***P < 0.001. **D**. Immunohistochemical staining of aPKCι, F4/80 and NF-κB protein in tumor tissue in various treatment groups. Scale bar, 200 μm. **E**. The structure and antitumor mechanisms of GEM-aPKCι-siRNA-L. **F**. Schematic illustration of the proposed Macrophages-aPKC_ɩ_-CCL5 feedback loop involved in the regulation of cholangiocarcinoma progression and improvement in chemotherapeutic responses

All liposomes were prepared as shown in Materials and Methods. Then, they were characterized to ensure that they qualified as liposomes. The key physicochemical characteristics of GEM-L and GEM-aPKCι-siRNA-L are shown in Supplementary Fig. 7. A soft agar growth assay was employed to evaluate the inhibitory effect of the various formulations. Compared to other treatment groups, the GEM-aPKCι-siRNA-L group showed significant inhibition of CCA cells anchorage-independent growth (Fig. 7A). To further evaluate the antitumor efficacy of the various formulations, we established a xenograft model of human CCA according to the schematic shown in Fig. 7B. Notably, consistent with the in vitro findings, Gem-aPKCι-siRNA-L-treated tumors had the smallest sizes among those of all treatment groups (Fig. 7C). To demonstrate the key role of aPKCι-siRNA in the inhibition of tumor growth, we sectioned the xenografts and analyzed the expression of aPKCι. We found that aPKCι-siRNA effectively interfered with aPKCι protein expression in vivo only when it was delivered effectively by liposomes. Furthermore, we observed a significant decrease in the number of F4/80^+^ macrophages when aPKCι was effectively knocked down (Fig. 7D). In addition, we found that treatment with GEM increased NF-κB expression (Fig. 7D). These data preliminarily suggest that the co-delivery of aPKCι-siRNA and GEM via liposomes produces enhanced antitumor effects in CCA.

Together, our results show that the Macrophages-aPKC_ɩ_-CCL5 feedback loop between mesenchymal-like cancer cells and TAMs promotes CCA progression and chemoresistance. Moreover, the liposome-encapsulated aPKCι-siRNA and GEM antagonized CCA GEM chemoresistance (Fig. 7E and F).

## Discussion

CCA is an aggressively invasive tumor with drug resistance and poor prognosis. The highly desmoplastic microenvironment of CCA presents an intricate immunological system [9]. Recent data have shown that cancer cells and infiltrating immune cells interact with each other by contacting or secreting cytokines to modulate the response to chemotherapy [38]. Understanding this interaction may offer new strategies for oncotherapy. Although CCA patients with TAM infiltration have poor long-term survival, the underlying mechanisms remain vague due to the limited number of studies. Thus, we investigated the crosstalk between CCA cells and TAMs and sought to identify a potential strategy for the remission of chemoresistance.

To study the mechanisms, we verified that increased expression of CD68 and CD206 in CCA tissue was positively correlated with poor outcomes. Several studies have demonstrated that aPKCι is involved in cell survival and plays a protective role against apoptotic stimuli [19, 39, 40]. Furthermore, NF-κB has been reported to be abnormally expressed in CCA cells and has been shown to be linked to malignant aggressiveness and chemoresistance through the release of proinflammatory cytokines [41, 42]. Notably, we found that the correlation between aPKCι and the expression of NF-κB, CD68, and CD206 is positive, and this biomarker combination is more effective than single biomarkers for predicting survival outcomes in patients with CCA. Thus, we hypothesized that aPKCι may play an essential role of shaping interactions between cancer cells and their associated macrophages via NF-κB signaling, which results in chemoresistance.

To further confirm our hypothesis, we constructed monocyte-derived macrophages (MDMs) generated from peripheral blood monocytes (PBMCs) and polarized them into M2 macrophages that are defined as TAMs in a variety of cancers [6, 43]. Here we found that TAMs induce GEM resistance by inhibiting apoptosis. A similar conclusion has been reported for pancreatic adenocarcinoma [44]. In the present study, we provide direct evidence for the activation of the NF-κB pathway by aPKC_ɩ_/P62 signaling in CCA. This result broadly confirms the work of other studies in this area linking aPKCι to P62 via the PB1 domain [29, 45].

The EMT process is executed in the tumor invasive front [14], where TAMs are usually located [46]. These reports suggest that cancer cells undergoing EMT may have advantages in shaping interactions between cancer cells and their associated macrophages. Indeed, our study found that TGFβ1 from TAMs induced EMT in CCA cells. Additionally, aPKC_ɩ_-siRNA treatment restrained NF-κB nuclear translocation and dampened the EMT response induced by TAMs. Subsequently, we selectively depleted macrophages in vivo, to demonstrate that TAMs play an important role in tumor progression. The use of CSF1R inhibitors is another appealing therapeutic strategy to target TAMs but has limited antitumor effects [47].

In turn, to further explore the mechanism of how CCA cells undergoing EMT regulate TAMs, we performed a cytokine array to identify cytokines secreted by mesenchymal-like cells overexpressing aPKC_ɩ_. Notably, among the cytokines, CCL5 was apparently induced. CCL5 is known to be a key factor in the tumor microenvironment, especially in recruiting monocytes and promoting macrophage function [8, 35, 36]. The experiments in vitro and vivo demonstrated that aPKC_ɩ_-CCL5-CCR5 axis recruits macrophages and programs their function of promoting tumor progression.

Collectively, these experiments demonstrate that TAMs induce aPKC_ɩ_ phosphorylation by producing TGFβ1, which induces EMT and chemoresistance. In turn, CCA cells undergoing EMT secrete CCL5, which recruits and activates macrophages, constituting a Macrophages-aPKC_ɩ_-CCL5 positive feedback loop. Consistent with our results, several positive feedback loops between cancer cells and TAMs, such as the GM-CSF-CCL18 loop, which promotes metastasis [48], and the CXCL1/2-S100A8/9 loop, which causes chemoresistance [49], have been reported. Currently, our understanding of the molecular mechanisms altering macrophage polarization is fragmented and incomplete. This is a meaningful topic for future research. Taken together, these data show that the Macrophages-aPKC_ɩ_-CCL5 loop could be a potential therapeutic target for CCA treatment, especially in cancers with high aPKC_ɩ_ expression levels.

Despite aPKC_ɩ_-siRNA exhibiting an anticancer effect in CCA cell lines, its therapeutic efficacy has not been assessed under translational medicine circumstances. Our data confirmed that the overexpression and activation of aPKC_ɩ_ can facilitate GEM resistance and cell survival in CCA. Thus, we sought to combine aPKC_ɩ_ siRNA and GEM to explore the anticancer effect. Due to the inefficient cell uptake and biological instability of nucleic acids, we designed liposomes that can simultaneously deliver aPKC_ɩ_-siRNA and GEM. Subsequently, we demonstrated that co-deliver of aPKC_ɩ_-siRNA and GEM by liposomes exhibits enhanced anti-tumor effects in vitro and in vivo. Unfortunately, because this is a preliminary investigation, we did not investigate the toxicity, pharmacokinetics, cellular uptake and biodistribution of the liposomes. In addition, previous studies have suggested that liposomes are associated with toxicity and inflammatory responses. Although assorted modifications have been attempted to achieve a balance between gene delivery toxicity and efficacy, vector-induced toxicity is still a challenge for nanoparticles. More broadly, research is still needed to improve the transfection efficiency and reduce toxicity. Notwithstanding these limitations, this study partially substantiates the rationality of the co-delivery of GEM and aPKC_ɩ_ siRNA since GEM-aPKC_ɩ_-siRNA-L presented enhanced synergistic antitumor effects in vivo. This exploration would be fruitful for further work to improve the anticancer effect in CCA.

## Conclusions

In summary, we demonstrated the prognostic significance of macrophages and aPKC_ɩ_ in CCA, and then identified the Macrophages-aPKC_ɩ_-CCL5 positive feedback loop in the tumor microenvironment that accelerates CCA progression and chemoresistance. Finally, we designed biocompatible liposomes to co-deliver GEM and aPKC_ɩ_-siRNA for CCA treatment and confirmed their synergistic antitumor effects. This thesis may deepen our understanding regarding the role of aPKC_ɩ_ in the CCA microenvironment and provides an important basis for the exploitation of new, effective therapeutic strategy for CCA.

## Supplementary Information

**Additional file 1.**

**Additional file 2.**

**Additional file 3.**

**Additional file 4.**

**Additional file 5.**

**Additional file 6.**

**Additional file 7.**

**Additional file 8.**

## Data Availability

The datasets generated and/or analyzed during the current study are not publicly available due to ethical reasons but are available from the corresponding author on reasonable request.

## Abbreviations

CCA: Cholangiocarcinoma
aPKC_ɩ_: Atypical protein kinase C iota
EMT: Epithelial-mesenchymal transition
TAMs: Tumor-associated macrophages
CM: Conditioned medium
GEM: Gemcitabine
TME: Tumor microenvironment
PBMCs: Peripheral blood monocytes
PDTC: Pyrrolidinedithiocarbamate ammonium
