# Supplementary material for: Macrophages-aPKCɩ-CCL5 Feedback Loop Modulates the Progression and Chemoresistance in Cholangiocarcinoma
Source: J Exp Clin Cancer Res. 2022 Jan 15;41:23. doi: 10.1186/s13046-021-02235-8 (PMC8760815; doi:10.1186/s13046-021-02235-8)
Supplement: Supplementary file 1 — Additional file 1. [file 13046_2021_2235_MOESM1_ESM.docx]

**Supplementary materials and methods**

1. ***10x Genomics single-cell sequencing technology***

Two paired tumor and paratumor samples from CCA patients were enrolled with informed consent in the study. Ethical approval was obtained from the Tongji Hospital Research Ethics Committee. All tissues were diagnosed by two independent pathologists and all patients had not received any preoperative adjuvant therapy. The clinicopathological information of these patients is shown in **Supplementary Table 1**.

First, these fresh tissues were disintegrated and dissolved into single cell suspension with a Human Tumor Dissociation Kit (Miltenyi Biotec GmbH). Then, mononuclear cells were isolated from the suspension by Percoll discontinuous density gradient centrifugal method and the cell viability was required to be >85% tested by an AO/PI double staining kit (Thermo Fisher Scientific).

Next, qualified immune cells were used to construct and sequence 5'gene expression library according to the 10x Genomics Chromium Single Cell V(D)J Reagent Kits User Guide. After Single-cell sequencing data were quality controlled and filtered, the STAR is used to compare the measured RNA sequence with the human GRCH38 reference genome to annotate the RNA sequence and obtain the single-cell transcription data matrix^1^. PCA and t-SNE dimensionality reduction analysis were performed on the single-cell transcription data matrix for cell cluster analysis and subtype division by Seurat v.3^2^. Based on the differentially expressed genes of each subtype, SingleR was used to identify and annotate cell type^3^.

1. ***Patients and specimens***

Paired human CCA tissues and paratumor tissues (2 cm distant from the tumor, n=70) were obtained during surgical resection at the Affiliated Tongji Hospital, Huazhong University of Science and Technology, China, between January 2014 and February 2019. Ethical approval was obtained from the Tongji Hospital Research Ethics Committee. All tissues were collected with informed consent and diagnosed by two independent pathologists. 40 patients had not received any adjuvant therapy and 30 patients had received postoperative adjuvant GEM-based therapy (1 months after surgery). The clinicopathological information of these patients is shown in **Supplementary Table 2**.

1. ***Cell lines***

Human CCA cell lines TFK-1 (purchased from American Type Culture Collection) and EGI-1 (purchased from The German Collection of Microorganisms and Cell Cultures GmbH) were used. Human peripheral blood CD14+ monocytes (hPBMCs) were isolated from healthy donors. All cells were grown according to standard protocols at 37 °C with 5% CO2.

1. ***Immunohistochemistry***

The expression levels of aPKCι, p-aPKCι, p65, CD68, CD206, F4/80 and CCR5 were detected by immunostaining, as previously described^4^. The process of evaluation was performed by three independent experienced investigators who were blinded to the patient conditions.

1. ***Immunofluorescence***

Immunofluorescence were performed as previously described^1^. Briefly, cells were incubated with primary antibodies against E-cadherin, vimentin and p65 followed by incubation with Secondary antibodies. And DAPI was used to stain cell nuclei. Images of these cells were observed and captured using a confocal microscopy (LSM880; Carl Zeiss, Oberkochen, Germany).

1. ***Lentiviral vector, plasmids and siRNA construction and transfection***

Lentiviral vectors containing human aPKCι-cDNA, aPKCι-siRNA, p62-cDNA and p62-siRNA were designed and purchased from Genechem Co., Ltd. (Shanghai, China). The pCDNA3.1-Flag-aPKCι, pCDNA3.1-Flag-aPKCι-D72A, pCDNA3.1-Flag-p62, pCDNA3.1-Flag-p62-K7A, pGL3-NF-κB-Luc, pGL3-CCL5-Luc, pGL3-CCL5(∆κB)-Luc, pGL3-κB-Re-Luc and pRL-TK renilla plasmids were designed and purchased from Tsingke Biological Technology Company (Beijing, China). Flag-tagged wild-type and site-specific mutants (aPKCι-D72A, P62-K7A) of these proteins based on previous research conclusions^5-7^. The siRNAs targeting aPKCι and p62 were designed and purchased from Ribobio (Guangzhou, China). The RNAi target sequences were listed in the **Supplementary Table 3**. All transfection was conducted according to the manufacturer’s instructions.

1. ***Quantitative real-time PCR***

Total RNA was extracted from frozen CCA specimens or cell lines by Trizol reagent (Invitrogen, USA). The complementary DNA was synthesized with PrimeScript^TM^ RT Master Mix (Takara Bio Inc, Dalian, China) according to the manufacturer’s instructions. Quantitative real-time PCR (qPT-PCR) was performed using a SYBR Premix EX Taq kit (Takara Bio Inc. Dalian, China) following the standard protocol. The results were normalized as previously described^4^. The primers used in this study are available in **Supplementary Table 3**.

1. ***Western blotting***

Western blotting (WB) was employed as previously described^1^. Cytoplasmic and nuclear proteins were isolated by a Nuclear and Cytoplasmic Protein Extraction Kit (Beyotime, Hangzhou, China) according to the manufacturer’s instructions. GAPDH and histone H3 were selected as the loading controls for cytoplasmic and nuclear fractions, respectively. The ImageJ2x analysis software package (National Institute of Mental Health, Bethesda, MD) was used to estimate the intensity of bands. The antibodies used in this study are shown in **Supplementary Table 4**.

1. ***Soft agar growth assay***

A soft agar growth assay was performed to detect anchorage-independent growth. Briefly, the bottom agar layer consisted of complete medium and 0.6% soft agar in 6-well plates. The cells were resuspended in medium supplemented with soft agar at a final concentration of 0.35%, plated on top of the bottom agar and cultured at 37°C in 5% CO_2_ for 3 weeks. Cell colonies were observed and quantified using an Olympus BX51 microscope. All experiments were independently repeated three times.

1. ***Conditioned medium***

Macrophages were washed with PBS when cells were 80% confluent. The cells were then incubated for another 24 hours in fresh serum-free medium. The supernatant of the medium was harvested and filtered and then used at a 30% final concentration to produce conditioned medium.

1. ***Cell proliferation and apoptosis assay***

CCA cells (1 × 10^4^) were plated in 96-well plates and allowed to adhere overnight. The medium was replaced with normal medium, MΦ-CM and M2-CM in the presence of different concentrations of GEM. After 48 hours of incubation, cell proliferation was detected by a Cell Counting Kit-8 (Dojindo Laboratories Co. Ltd., Kumamoto, Japan). After incubation for 2 hours at 37 °C, the absorbance was measured by a plate reader (Bio-Tek Instruments, VT, USA) at 450 nm according to the standard protocol. All samples were analyzed in triplicate.

For the apoptosis assay, CCA cells were incubated in normal medium, MΦ-CM and M2-CM in the presence or absence of 10 M GEM. After 48 hours, the cells were harvested using trypsin without EDTA, washed twice with PBS, stained with Annexin V-FITC/propidium iodide (MBL, Nagoya, Japan), and analyzed by flow cytometry (BD Biosciences, Franklin Lakes, NJ, USA).

1. ***Luciferase reporter assay***

The transfected cells were lysed and the resulting lysates were centrifuged by an Eppendorf microcentrifuge. Luciferase and renilla signals were assayed using a Dual-Luciferase Reporter Assay Kit (Beyotime, Hangzhou, China). Firefly luciferase activity was normalized to renilla luciferase activity.

1. ***Flow cytometry***

Flow cytometry was used to detect the biomarkers of macrophage differentiation and to analyze the percent of apoptotic cells. All experiments were repeated independently at least three times. The main antibodies are detailed in the **Supplementary Table 5.**

1. ***Peripheral blood monocyte isolation and differentiation***

Human peripheral blood CD14^+^ monocytes were isolated from leukocyte cones obtained from healthy donors by high-gradient magnetic sorting using anti-CD14 microbeads (Miltenyi Biotec, Shanghai, China).

For differentiation into M2 macrophages, peripheral blood monocyte were incubated with 150 nM PMA for 24 hours and subsequently with 100 ng/mL of IL-4 and 100 ng/mL of IL-13 for 48 hours. For M1 macrophages, PMA-treated peripheral blood monocyte were stimulated with 20 pg/mL lipopolysaccharide (LPS) and 100 ng/mL IFN-γ for 48 hours. The biomarkers of macrophage differentiation were confirmed by flow cytometry, as described above.

1. ***Transwell*** ***migration and invasion assay***

Transwell chambers (Corning, NY, USA) were used to estimate cell invasion. The transwell chambers were precoated with Matrigel (BD Biosciences, NJ, USA) and RPMI-1640 (1:8). 1x10^5^ cells per chamber were placed into upper chamber containing 200ul serum-free medium for 48 hours. And the lower chamber was supplied with 500ul complete culture medium. Then, cells at lower surface of the filters were stained and counted using Image-Pro Plus v6.0 software package (Media Cybernetics Inc., MD, USA). For migration assay, the migration of monocytes was measured with a 5 μm transwell chamber. The monocytes were seeded into the upper chamber, and CCA cells treated as Figure 5D were cultured in the lower chamber. The number of cells on the lower side of the membrane was scored per field of view following 24 hours. All experiments were repeated at least three times.

1. ***Immunoprecipitation***

CCA cells were transfected with the indicated expression plasmids and immunoprecipitated as described previously^4^. Briefly, the cells were lysed by RIPA buffer and divided into parallel groups named IPs or input. Then, the primary antibody or IgG was added into the lysates for incubating overnight on a spinning wheel at 4℃. The input was used as a positive control. Subsequently, the mixture was incubated with Protein A + G agarose beads at 4°C for 3 h. The beads were collected after centrifugation and washed 5 times with RIPA buffer. The immunoblotting was performed with the indicated antibodies correspondingly. The immunoprecipitation of CCA tissues was consistent with above approach. All assays were repeated at least three times.

1. ***Tumor-derived cytokine screening***

CM was collected from mesenchymal-like TFK-1 cells or negative control cells. Multiple cytokine expression levels in CM were quantified using a Human Inflammation Array G3 (G-Series, AAH-INF-G3-4, Ray Biotech, Inc., Norcross, GA) according to the manufacturer’s instructions. The array was scanned and quantified using GenePix 4000B and GenePix Pro 6.0 (Axon Instruments, USA). The positive controls on each subarray were used to normalize the intensity being compared on different subarrays.

1. ***Enzyme-linked immunosorbent assay***

TGFβ1 and CCL5 ELISA kits were purchased from Boster, and ELISAs were performed as described previously^4^.

1. ***Preparation of GEM/siRNA-L nanoliposomes***

The liposomes were synthesized according to a previous study using the thin-film dispersed hydration method. The lipid composition of GEM liposomes (GEM-Ls) was DOTAP/CHOL/MPEG-DSPE2000 at a molecular ratio of 40:55:5 (at weights of 28, 21.3 and 14.7). The lipid was dissolved in chloroform and then dried to form a thin lipid film at a temperature of 40 °C using a rotary evaporator under reduced pressure. GEM dissolved in 4 mL PBS (pH 6.8) was used to hydrate the resulting lipid film at 60 °C for 30 min to form preliminary liposomes. The large multi-unit liposomes were extruded through a polycarbonate membrane (0.2 μm pore size) 12 times to obtain nanosized and unilamellar liposomes. Negatively charged siRNA was added to the positively charged liposomes for adsorption by electrostatic interaction at a w/w (weight liposome/weight siRNA) ratio of more than 200:1 in RNase-free H2O to form the final GEM/aPKCι-siRNA-L liposome complex.

1. ***Characterization of GEM/siRNA-L nanoliposomes***

The physicochemical characteristics of the developed nanoparticles were determined using a Zeta PALS instrument (DLS, Zeta Plus, Brookhaven Instruments, USA) conforming to the manufacturer’s specification. Each sample was detected 3 times at room temperature, and the standard deviations and average values were calculated.

1. ***Animal studies***

For the antitumor research, the establishment of xenograft models was performed as previously described. Briefly, subcutaneous xenografts were formed in the front armpit of four-week-old female BALB/C nude mice by injecting EGI-1 cells (2×10^6^ cells, suspended in 150μl of PBS, 5 mice/group). When the tumor volume reached approximately 50 mm^3^, the different indicated drug formulations (50 mg/kg GEM, 10nmol/mouse siRNA) were injected through the tail vein once a week for a total of 4 doses. All mice were monitored twice a week and sacrificed 4 weeks later. The tumor volume was calculated by the equation V = length× width^2^ × 0.5.

For TAMs depletion and recruitment experiments, the subcutaneous xenograft models were established as described above. Briefly, CCA cells transfected with aPKCι-cDNA or a control vector were injected subcutaneously into the front armpit region of BALB/c nude mice (6 mice/group). For the TAMs depletion experiment, after one week, xenografted mice were intravenously injected with clodronate liposomes at 4 μl/g (ClodronateLiposomes.org). The control for this treatment was PBS liposomes. All of these treatments were given twice a week for 3 weeks. For the TAM recruitment experiment, one week after inoculation, mice were treated every day for 3 weeks by oral gavage (100 µl/mouse). Maraviroc (5 mg/kg) was dissolved in DMSO and diluted with saline. The final concentration of DMSO in all groups was 0.5%. All mice were euthanized 4 weeks later.

The lung metastasis model was established by injecting EGI-1 cells transfected with aPKCι-cDNA into the tail veins of mice (1× 10^6^ cells suspended in 100μl of PBS). All mice were randomized into 2 groups (6 mice/group) after one week. Subsequently, the intravenous injection of clodronate liposomes at 4 µl/g was performed twice a week. PBS liposomes were used as the treatment in the control group. All mice were euthanized 6 weeks after treatment. The number of lung metastases was counted under a microscope.

All animal experiments were performed under specific pathogen-free (SPF) conditions in the Central Animal Laboratory of Tongji Medical College. The protocol was approved by the Committee on the Ethics of Animal Experiments of Tongji Medical College, HUST.

***Statistical analysis***

Statistical analysis was performed using SPSS 23.0 software. Quantitative data are presented as the mean ± SD. The signiﬁcance of differences for different groups was determined by using two-tailed Student’s t test, analysis of variance or Pearson’s correlation test. Clinical relevance were evaluated using the χ^2^ test, and the survival among subgroups was assessed by Kaplan-Meier curves and log-rank test. The Cox proportional hazards model was used to perform univariate and multivariate analyses. P < 0.05 was considered statistically signiﬁcant. (*P < 0.05; **P < 0.01; ***P < 0.001; ****P < 0.0001.)

References

1. Vallejo AF, Davies J, Grover A, et al. STAR: ultrafast universal RNA-seq aligner. Bioinformatics. 2013 Jan 1;29(1):15-21.
2. Butler A, Hoffman P, Smibert P, et al. Integrating single-cell transcriptomic data across different conditions, technologies, and species. Nat Biotechnol. 2018 Jun;36(5):411-420.
3. Aran D, Looney AP, Liu L, et al. Reference-based analysis of lung single-cell sequencing reveals a transitional profibrotic macrophage. Nat Immunol. 2019 Feb;20(2):163-172.
4. Qian YW, Yao W, Yang T, et al. aPKC-iota/P-Sp1/Snail signaling induces epithelial-mesenchymal transition and immunosuppression in cholangiocarcinoma. Hepatology 2017;66:1165-1182.
5. Hirano Y, Yoshinaga S, Ogura K, et al. Solution structure of atypical protein kinase C PB1 domain and its mode of interaction with ZIP/p62 and MEK5. J Biol Chem 2004;279:31883-90.
6. Wilson MI, Gill DJ, Perisic O, et al. PB1 domain-mediated heterodimerization in NADPH oxidase and signaling complexes of atypical protein kinase C with Par6 and p62. Mol Cell 2003;12:39-50.
7. Lamark T, Perander M, Outzen H, et al. Interaction codes within the family of mammalian Phox and Bem1p domain-containing proteins. J Biol Chem 2003;278:34568-81

**Supplementary Table 1. Clinicopathological characteristics of CCA patients involved in the 10x Genomics single-cell sequencing technology**

| **Patient ID** | **#1** | **#2** |
| --- | --- | --- |
| **Age (year)** | 71 | 55 |
| **Gender** | male | male |
| **Differentiation** | well | poor |
| **Stage (AJCC 8^th^ Edition)** | T2N1M0 | T3N1M0 |

**Supplementary Table 2. Clinicopathological characteristics of CCA patients.**

|  | **Cholangiocarcinoma (n = 70)** | |
| --- | --- | --- |
| **Characteristics** | **number** | **Percentage** |
| **Age (y)** |  |  |
| ≤ 60 | 27 | 38.6 |
| > 60 | 33 | 61.4 |
| **Gender** |  |  |
| male | 36 | 51.4 |
| female | 34 | 48.6 |
| **Differentiation** |  |  |
| well | 27 | 38.6 |
| moderately/poorly | 43 | 61.4 |
| **Nodal invasion** |  |  |
| Negative | 45 | 64.3 |
| Positive | 25 | 35.7 |
| **TNM stage** |  |  |
| I - II | 33 | 47.1 |
| III - IV | 37 | 52.9 |

**Supplementary Table 3. Primers and RNAi target sequences.**

| **Target sequences** | | |
| --- | --- | --- |
| **Name** | **sequences** | |
| aPKCι | TTTAGACTTTATGAGCTAA | |
| aPKCι | CCTGAAGAACATGCCAGATTT | |
| P62 | GCATTGAAGTTGATATCGAT | |
| **PCR Primers** | | |
| **Name** | **Forward primer** | **Reverse primer** |
| CD80 | ATCTGACGAGGGCACATACG | TCACTTCAGCCAGGTGTTCC |
| CD206 | CCGACCCTTCCTTGACTAATCC | GTCTCCGCTTCATGCCATTG |
| IL-10 | CCCACTTCCCAGGCAACC | ACCCTTAAAGTCCTCCAGCAAG |
| IL-12 | TCACAAAAGATAAAACCAGCACAGT | GCCAGGCAACTCCCATTAGTT |
| GAPDH | GGTCGGAGTCAACGGATTTG | GGAAGATGGTGATGGGATTTC |
| TGFβ1 | GCAACAATTCCTGGCGATACC | TCCACGGCTCAACCACTG |
| CCL5 | GCCTGTTTCTGCTTGCTCTTG | TGTAACTGCTGCTGTGTGGTAG |
| NF-κB | TTTTGGTGTCCTTGGGTGCT | GACATCAGCCCCACACTTCA |
| CD68 | CAGGGAATGACTGTCCTCACA | CTCTCTGTAACCGTGGGTGT |
| aPKCι | CACACTTTCCAAGCCAAGCG | GGCGTCCAAGTCCCCATATT |

**Supplementary Table 4. Information of antibodies used in this study**

| **Name** | **WB** | **IHC** | **IF** | **Company** | **Cat. No.** |
| --- | --- | --- | --- | --- | --- |
| aPKCι | 1: 500 | 1: 200 | 1:50 | ProteinTech Group | 13883-1-AP |
| P-aPKCι | 4ug/ml | 1:50 | / | Invitrogen | 700582 |
| NF-κB | 1: 1000 | 1: 200 | 1:50 | ProteinTech Group | 10745-1-AP |
| p-NF-κB | 1:10000 | / | / | Abcam | ab76302 |
| p62 | 1:1000 | / | / | ProteinTech Group | 18420-1-AP |
| p62 | 1:1000 | / | / | CST | 88588S |
| CD68 | 1: 300 | / | / | ProteinTech Group | 25747-1-AP |
| CD68 | 0.5ug/ml | 0.5ug/ml | / | Abcam | ab125212 |
| CD68 | / | 1:5 | / | Zsbio | ZM-0060 |
| CD206 | 1: 1000 | / | / | ProteinTech Group | 18704-1-AP |
| CD206 | 1:1000 | 1:400 | / | CST | 91192 |
| GAPDH | 1: 1000 | 1:250 | / | Abcam | ab8245 |
| Histone-H3 | 1: 1000 | 1:50 | / | ProteinTech Group | 17168-1-AP |
| cleaved caspase-3 | 1:1000 | / | / | CST | 9661 |
| Flag | 1:1000 | / | 1:50 | CST | 8146T |
| E-cadherin | 1:1000 | / | 1:50 | CST | 14472 |
| E-cadherin | 1:1000 | / | 1:25 | ProteinTech Group | 20874-1-AP |
| Vimentin | 1:1000 | / | 1:50 | ProteinTech Group | 10366-1-AP |
| F4/80 | 1:1000 | 1:250 | / | CST | 70076 |
| AKT | 1:1000 | / | / | CST | 2920 |
| p-AKT | 1:2000 | / | / | CST | 4060 |
| STAT3 | 1:1000 | / | / | ProteinTech Group | 10253-2-AP |
| p-STAT3 | 1:2000 | / | / | Abcam | ab76315 |
| CCR5 | 1:500 | / | / | Servicebio | GB11505 |
| **Name** | **Neutralization** | | | **Company** | **Cat. No.** |
| CCL5 | 0.2ug/ml | | | R&D System | MAB678-SP |
| TGFβ1 | 0.4ug/ml | | | R&D System | MAB678-SP |
| **Flow cytometry** | | | | **Company** | **Cat. No.** |
| APC Mouse Anti-Human CD206 | | | | BD Pharmingen | 561763 |
| PE Mouse Anti-Human CD80 | | | | BD Pharmingen | 560925 |
| BB515 Rat Anti-CD11b | | | | BD Pharmingen | 564455 |
| PerCP Mouse Anti-Human CD14 | | | | Biolegend | 325631 |

**Supplementary** **Figure Legends**

**Supplementary Figure 1 The clinical signiﬁcance of macrophages infiltration and aPKCι in human CCA**

1. The protein expression of aPKCι and macrophage markers (CD68, CD80 and CD206) in representative CCA samples (T) and pair-matched paratumor tissues (P) evaluated by western blotting.
2. The mRNA expression of aPKCι and macrophage markers (CD68, CD80 and CD206) in representative CCA samples (T) and pair-matched paratumor tissues (P) evaluated by qPCR.
3. Linear regression was used to analyze the correlations between aPKCι with CD68, CD80 and CD206 respectively.

**Supplementary Figure 2** **M2 macrophages induce aPKCι-mediated CCA cell chemoresistance to GEM**

1. Overall survival rates of CCA patients treated with GEM-based chemotherapy or not after surgery were compared using Kaplan-Meier analysis.
2. Schematic of the protocol for macrophage differentiation from PBMCs.
3. Flow cytometry detection of CD11b, CD80 and CD206 in MΦ, M1 and M2 macrophages.
4. mRNA expression of CD80, CD206, IL-10 and IL-12 in MΦ, M1 and M2 macrophages quantified by qRT-PCR.
5. The protein and mRNA expression of aPKCι in the indicated CCA cells evaluated by western blotting and qPCR, respectively.
6. IC50 of gemcitabine in the indicated cells. Each bar represents the mean ±SD of three independent experiments.
7. Annexin V-FITC and PI staining of the indicated cells treated without gemcitabine. Each bar represents the mean ± SD of three independent experiments.

**Supplementary Figure 3 aPKCι mediates NF-κB activation to contribute to M2 macrophages-induced chemoresistance**

1. WBs were used to detect the expression of p-aPKCι, P62, p-NF-κB and test the NF-κB (p65) nuclear translocation in the indicated cells. CCA cells were transfected with the empty vector as a negative control (NC) and cells without any treatment were used as blank control (aPKCι).
2. Anchorage-independent growth was evaluated by soft gar assays in the indicated CCA cells treated with gemcitabine (10 μM) for 48 hours.
3. Kaplan−Meier analysis indicating the correlation between overall survival in CCA patients with co-expression of nuclear NF-κB and CD206.
4. A Co-IP assay was performed to detect the interaction between aPKCι and P62 in CCA cell lines (upper). Effect of aPKCι PB1 mutations (bottom), CCA cells were transfected with a Flag-aPKCι (aPKCι wild type) plasmid, Flag-aPKCι-D72A plasmid or Flag-empty vector (used as a negative control). After 24 hours, cell extracts were immunoprecipitated with an anti-P62 antibody, and the immunoprecipitates were analyzed by immunoblotting with anti-aPKCι, anti-Flag and anti-P62 antibodies.
5. WBs for aPKCι, p-aPKCι, P62, p-NF-κB, and NF-κB of CCA cells after transfection with or without P62-WT, P62-K7A or P62-siRNA.
6. NF-κB luciferase reporter activity in CCA cells after treatment as described in E.

**Supplementary Figure 4** **M2 macrophages derived TGFβ1 induce CCA cell EMT via the aPKCι and NF-κB activation**

1. TGFβ1 expression in MΦ/M1/M2 macrophages was determined by qRT-PCR. ELISA showing the levels of TGFβ1 in the above macrophage populations.
2. Confocal fluorescence microscopy of p65/DAPI staining in EGI-1 cells treated with or without TGFβ1 together with PDTC or si-aPKCι#1 treatment. Scale bar, 20 μm.
3. WBs were used to detect the expression of EMT markers (E-cadherin and Vimentin) in the indicated cells.
4. Immunofluorescence were used to detect E-cadherin and vimentin expression in EGI-1 cells treated with or without CM from MΦ macrophages (MΦ-CM), M2-CM alone or with LY2157299, an anti-TGFβ1 neutralizing antibody, PDTC, or recombinant human TGFβ1. Scale bar, 20 μm.
5. Wound healing and invasion assays of EGI-1 cells treated as described in (D). Scale bar, 200 μm (mean ± SD, n = 3; *P < 0.05, **P < 0.01, ***P < 0.001 and ****P < 0.0001; P values were obtained using two-tailed Student’s t tests).

**Supplementary Figure 5 CCL5 secreted by aPKCι-induced mesenchymal-like CCA cells mediates the chemotactic migration and activation of macrophages**

1. Flow cytometry for expression of CD80/CD206 in macrophages treated with CM from mesenchymal-like TFK-1 cells. The CM of TFK-1 cells transfected with empty vector was used as a negative control.
2. Migration assay in CD14^+^ monocytes stimulated with or without CM from TFK-1 cells transfected with an empty vector (negative control, NC-CM), CM from mesenchymal-like TFK-1 cells (transfected with aPKCι-cDNA). Scale bar, 200μm.
3. WBs were used to detect the expression of CCL5 in the indicated cells.
4. Schematic structure of the human CCL5 promoter. Sequences of the wild-type and mutated CCL5 promoters used in this study
5. qRT-PCR was employed to detect the expression of IL12/TGF-β1 in macrophages treated with or without control TFK-1-CM, CM from mesenchymal-like TFK-1 cells (TFK-1^M^-CM) alone or with an anti-CCL5 neutralizing antibody, PDTC, or recombinant human CCL5.
6. WBs were employed to analysis the phosphorylation levels of AKT, NF-κB, and STAT3 in macrophages treated as described in (D).

**Supplementary Figure 6 The macrophage-aPKCι-CCL5 feedback loop promotes CCA growth and metastasis in vivo**

1. The volume (upper) and representative images (bottom) of TFK-1 xenograft tumors are shown from two different groups treated with clodronate liposomes or control liposomes.
2. Representative images from TFK-1 tumor sample serial sections stained for aPKCι, p-aPKCι and F4/80 are shown. Scale bar, 200 μm.
3. The volume (upper) and representative images (bottom) of TFK-1 xenograft tumors are shown from three different groups as indicated.
4. Representative images from TFK-1 xenograft tumors serial sections stained for aPKCι, CCR5, p-aPKCι and F4/80 are shown. Scale bar, 200 μm.

**Supplementary Figure 7 Co-delivery of aPKCι-siRNA and GEM via liposomes for the effective treatment of CCA**

1. Key parameters of GEM-L and GEM-siRNA-L liposomes. Data are expressed as mean ± SD of three independent samples.
