# Supplementary figures and images for: Macrophages-aPKCɩ-CCL5 Feedback Loop Modulates the Progression and Chemoresistance in Cholangiocarcinoma
Source: J Exp Clin Cancer Res. 2022 Jan 15;41:23. doi: 10.1186/s13046-021-02235-8 (PMC8760815; doi:10.1186/s13046-021-02235-8)

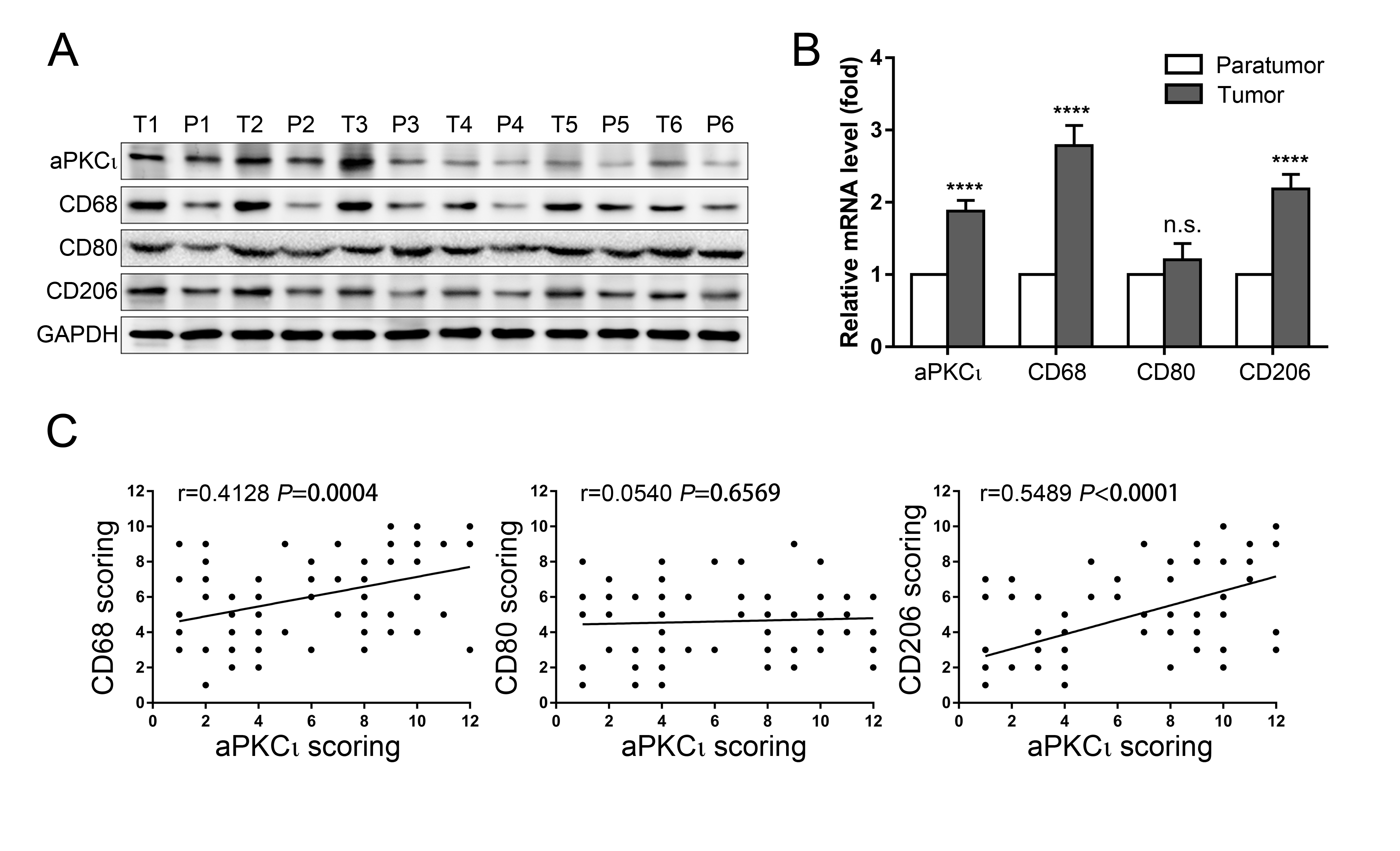

Supplement: Supplementary file 2 — Additional file 2. [file 13046_2021_2235_MOESM2_ESM.tif]

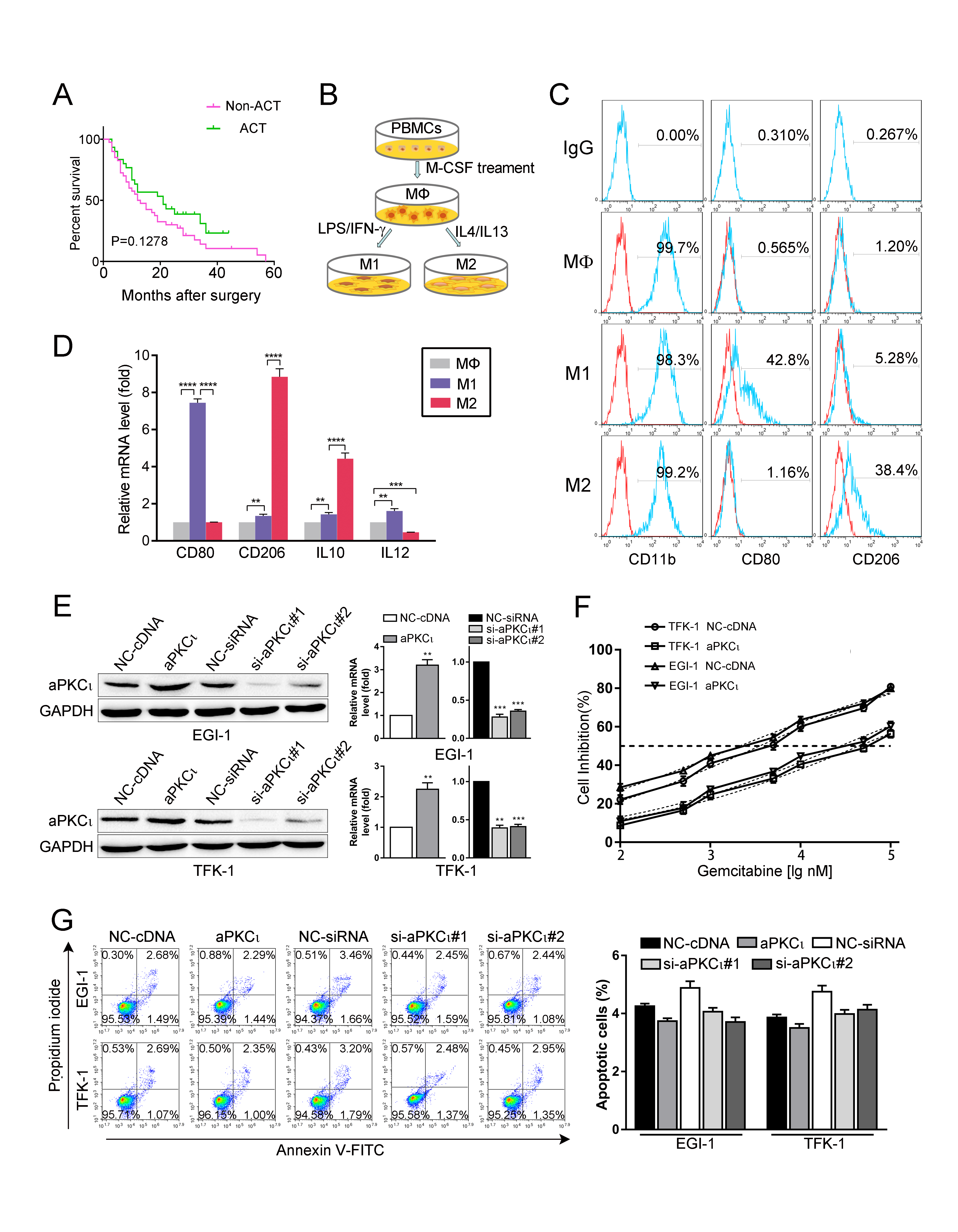

Supplement: Supplementary file 3 — Additional file 3. [file 13046_2021_2235_MOESM3_ESM.tif]

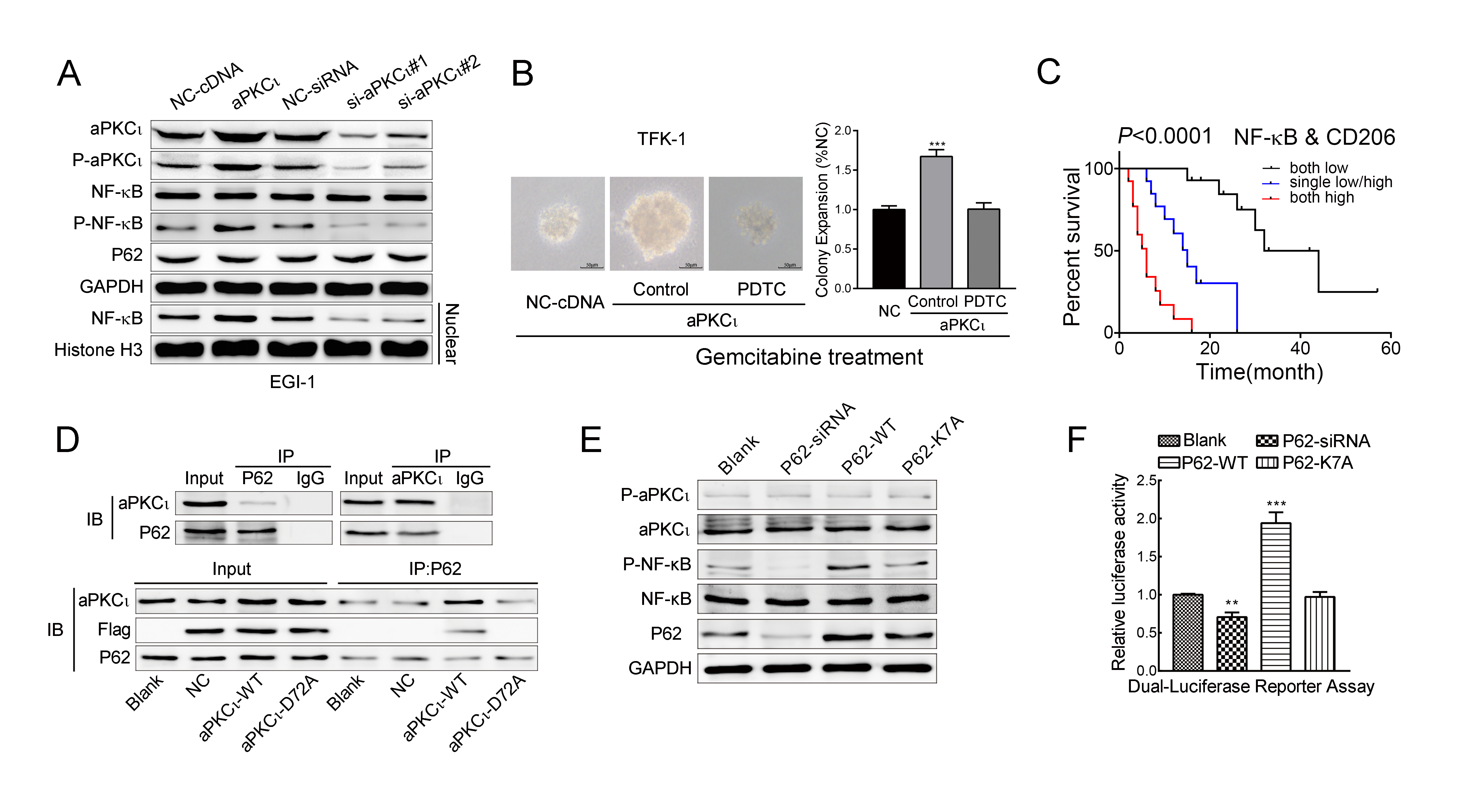

Supplement: Supplementary file 4 — Additional file 4. [file 13046_2021_2235_MOESM4_ESM.tif]

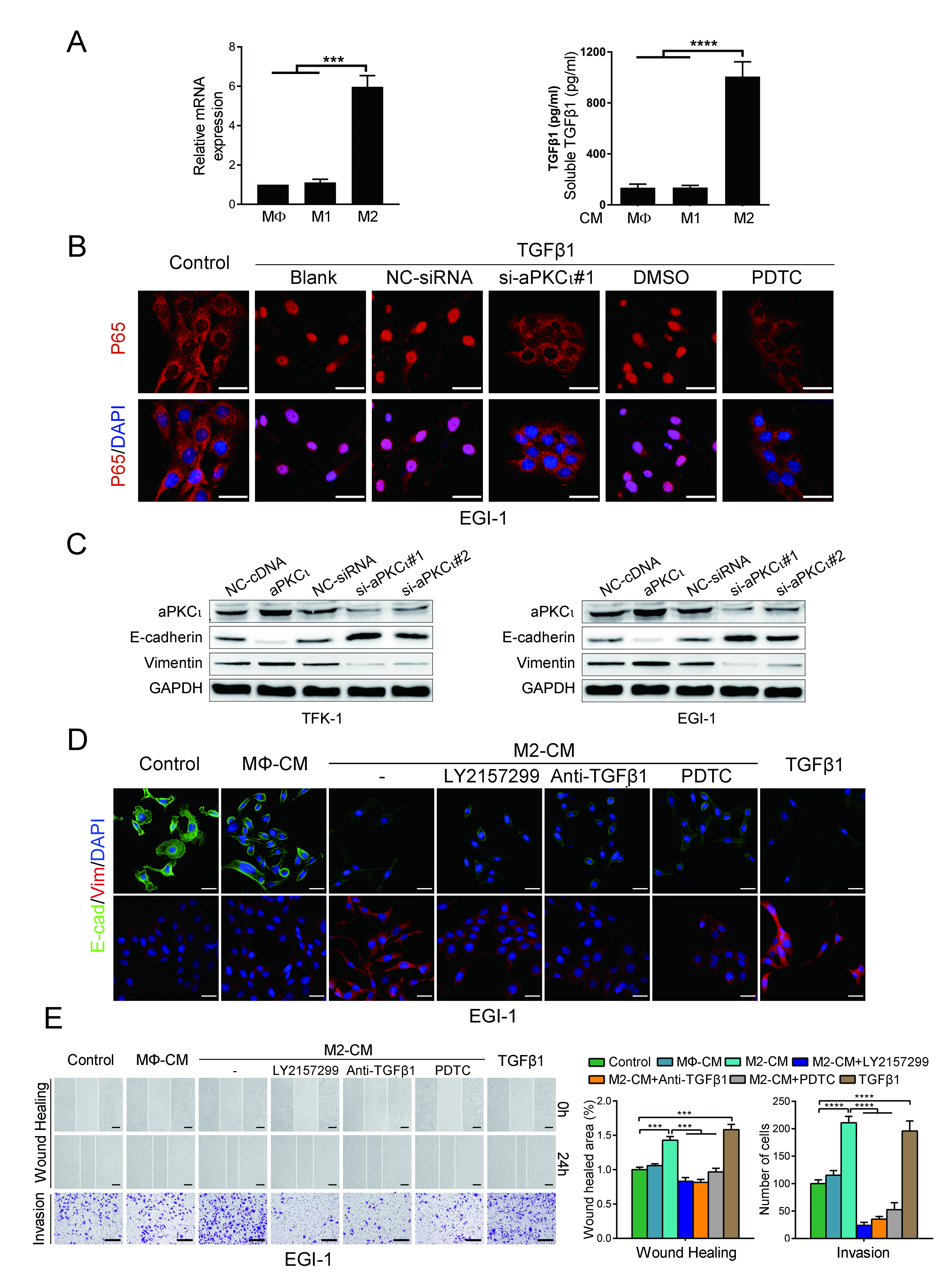

Supplement: Supplementary file 5 — Additional file 5. [file 13046_2021_2235_MOESM5_ESM.tif]

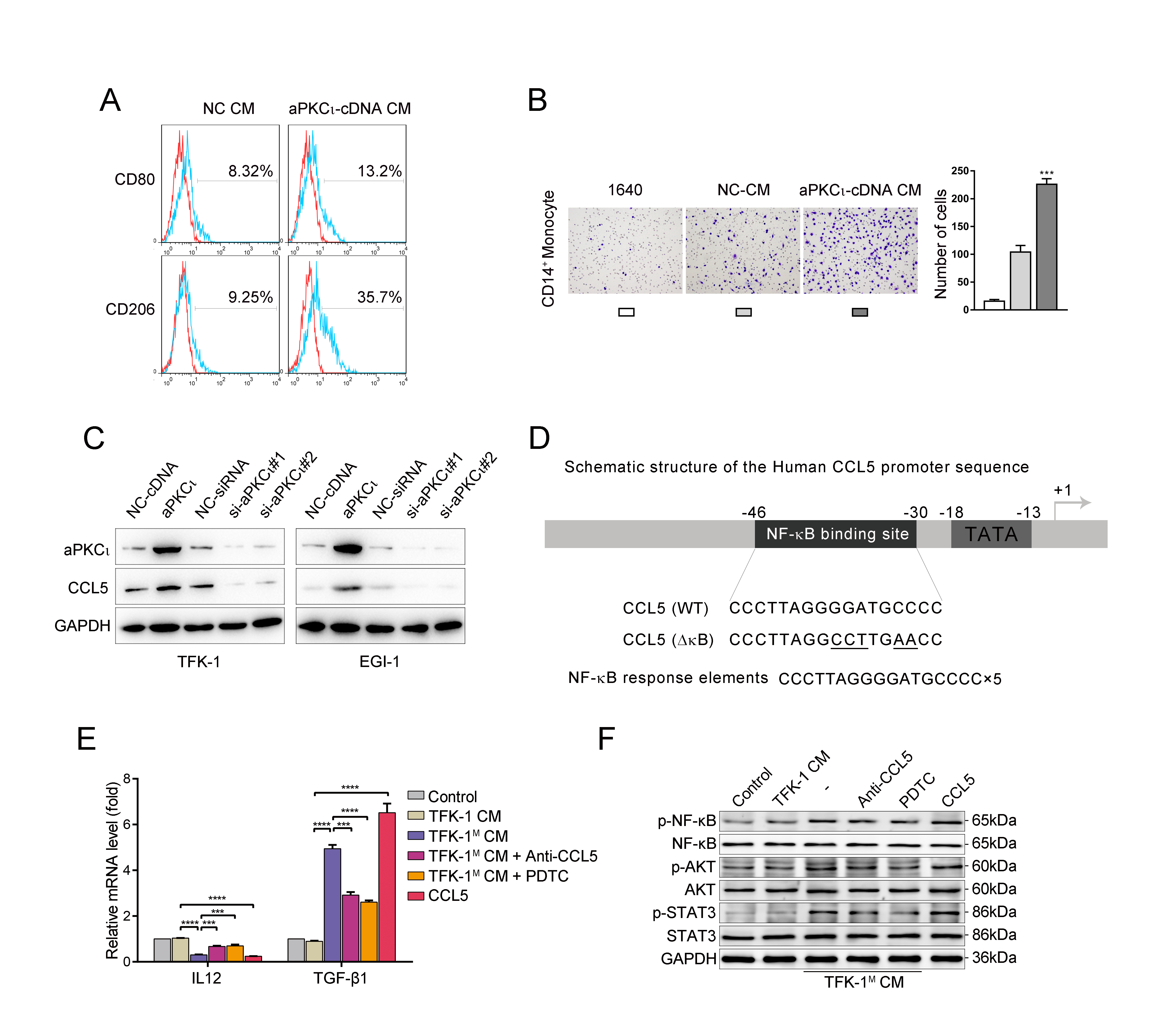

Supplement: Supplementary file 6 — Additional file 6. [file 13046_2021_2235_MOESM6_ESM.tif]

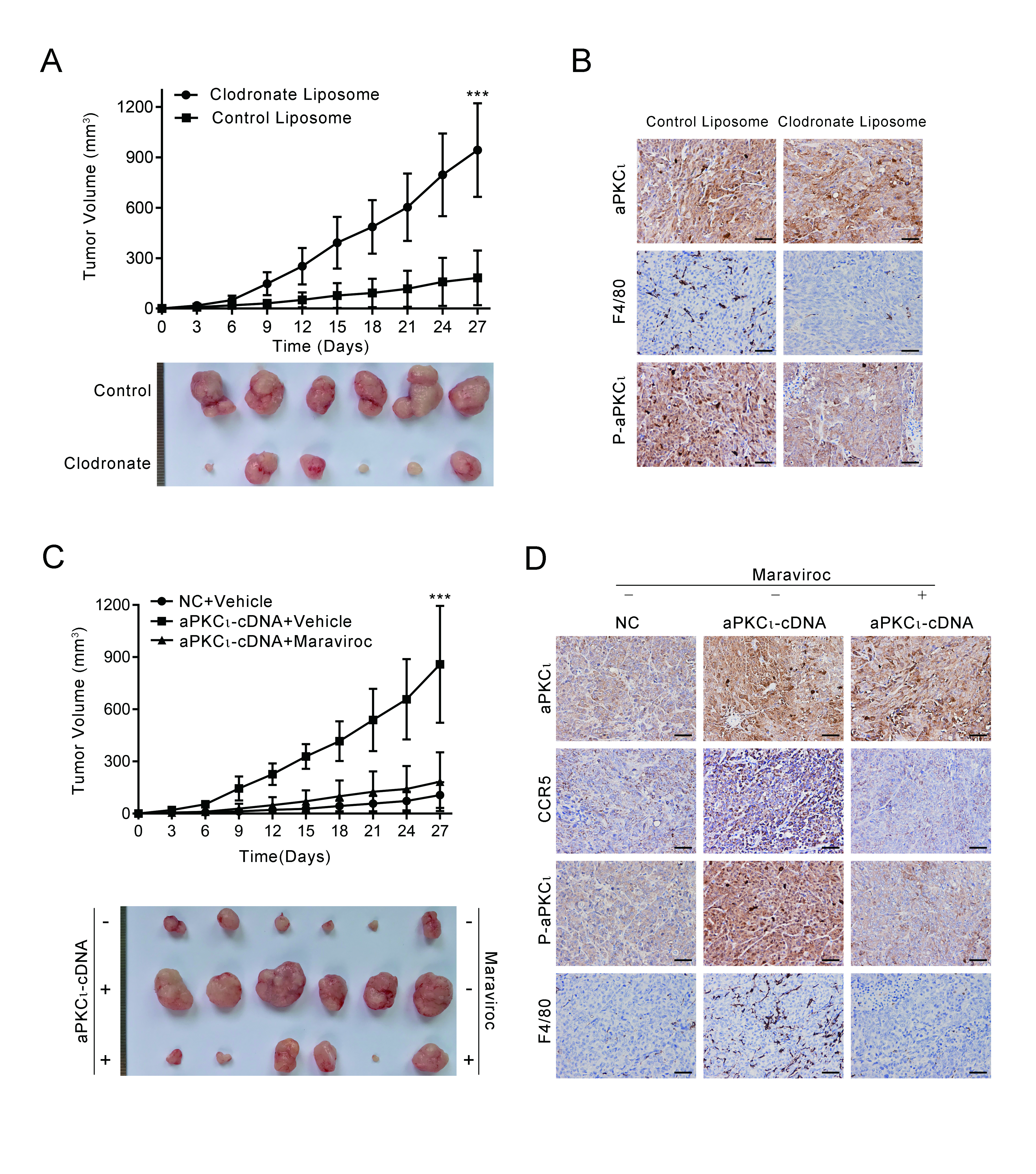

Supplement: Supplementary file 7 — Additional file 7. [file 13046_2021_2235_MOESM7_ESM.tif]

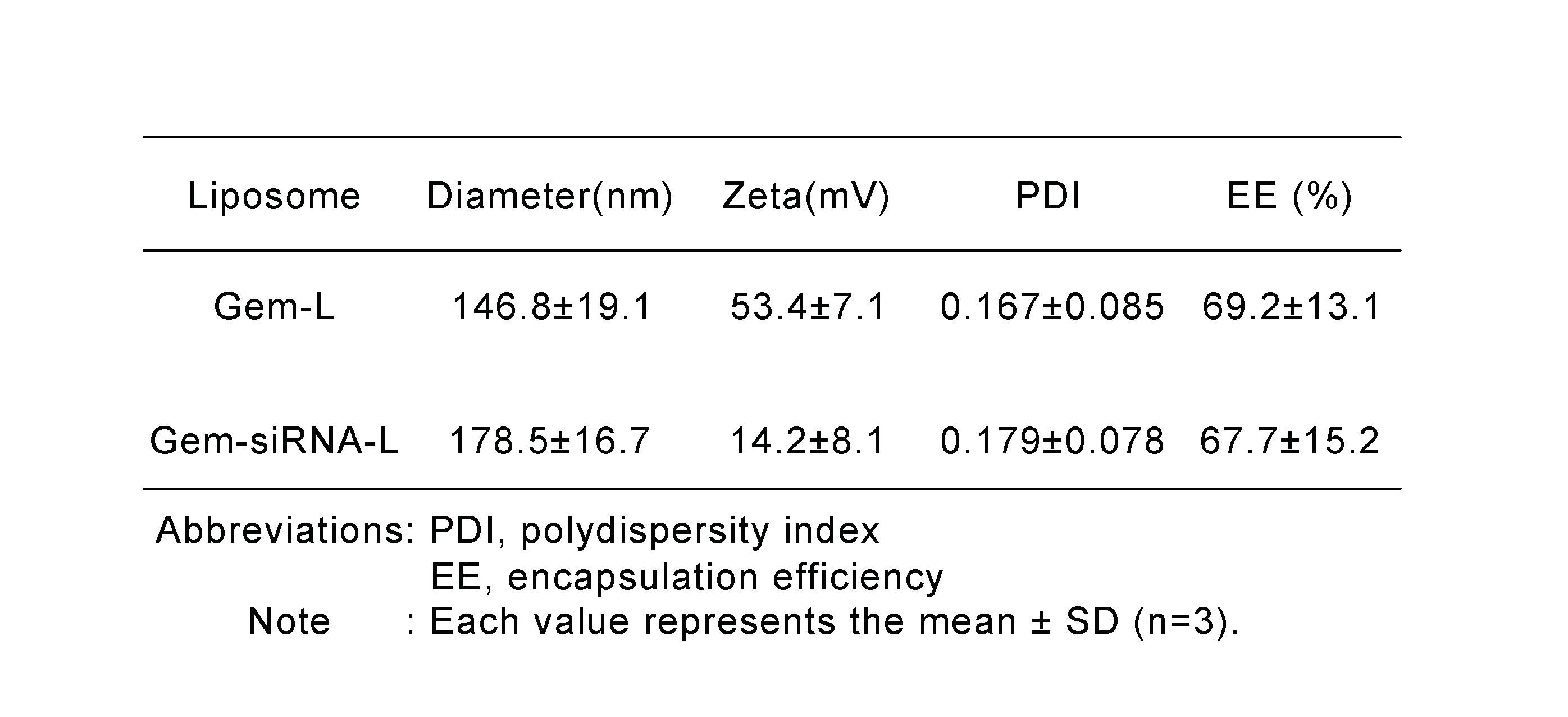

Supplement: Supplementary file 8 — Additional file 8. [file 13046_2021_2235_MOESM8_ESM.tif]
